# Stomata on the abaxial and adaxial leaf surfaces contribute differently to leaf gas exchange and photosynthesis in wheat

**DOI:** 10.1111/nph.18257

**Published:** 2022-06-30

**Authors:** Shellie Wall, Silvere Vialet‐Chabrand, Phillip Davey, Jeroen Van Rie, Alexander Galle, James Cockram, Tracy Lawson

**Affiliations:** ^1^ School of Life Sciences University of Essex Colchester CO4 3SQ UK; ^2^ BASF BBCC – Innovation Center Gent Technologiepark‐Zwijnaarde 101 9052 Ghent Belgium; ^3^ NIAB 93 Lawrence Weaver Road Cambridge CB3 0LE UK

**Keywords:** bean (*Phaseolus vulgaris* L.), bread wheat (*Triticum aestivum* L.), net CO_2_ assimilation rate (*A*), split‐chamber cuvette, stomatal conductance (*g*_s_), stomatal density

## Abstract

- Although stomata are typically found in greater numbers on the abaxial surface, wheat flag leaves have greater densities on the adaxial surface. We determine the impact of this less common stomatal patterning on gaseous fluxes using a novel chamber that simultaneously measures both leaf surfaces.
- Using a combination of differential illuminations and CO_2_ concentrations at each leaf surface, we found that mesophyll cells associated with the adaxial leaf surface have a higher photosynthetic capacity than those associated with the abaxial leaf surface, which is supported by an increased stomatal conductance (driven by differences in stomatal density).
- When vertical gas flux at the abaxial leaf surface was blocked, no compensation by adaxial stomata was observed, suggesting each surface operates independently. Similar stomatal kinetics suggested some co‐ordination between the two surfaces, but factors other than light intensity played a role in these responses.
- Higher photosynthetic capacity on the adaxial surface facilitates greater carbon assimilation, along with higher adaxial stomatal conductance, which would also support greater evaporative leaf cooling to maintain optimal leaf temperatures for photosynthesis. Furthermore, abaxial gas exchange contributed *c.* 50% to leaf photosynthesis and therefore represents an important contributor to overall leaf gas exchange.

## Introduction

Stomata are microscopic structures found over the predominantly waterproof and CO_2_ impermeable leaf cuticle, comprising two specialized guard cells surrounding a central pore, which adjust in size to control diffusional gaseous flux between the interior of the leaf and the atmosphere (Zeiger *et al*., 1987; Lawson & Weyers, 1999; Hetherington & Woodward, 2003). For plants to function efficiently, stomata open and close in response to various external and internal stimuli to carefully balance CO_2_ uptake and maintain photosynthetic carbon assimilation (*A*) with water loss via transpiration (*E*). Stomatal conductance (*g*_s_), a measure of the ease with which gases are exchanged through stomata, is commonly used to assess stomatal behaviour and functional responses to different environmental conditions. High *g*_s_ enables CO_2_ uptake for photosynthetic carbon assimilation (*A*) but is also associated with high water loss through transpiration, with implications for plant water status (Lawson & Vialet‐Chabrand, 2019). However, transpirational water loss also facilitates nutrient uptake and is essential for maintaining an appropriate leaf temperature for optimal photosynthesis, particularly under conditions of high light intensity that drive high photosynthesis (Willmer & Fricker, 1996; Shimazaki *et al*., 2007; Morison *et al*., 2008; Lawson *et al*., 2010; Lawson & Blatt, 2014).

Stomatal conductance is determined by anatomical characteristics, including stomatal density (SD), size (guard cell length; GCL) and patterning, as well as by functional responses that alter pore aperture (Willmer & Fricker, 1996; Weyers & Lawson, 1997; Hetherington & Woodward, 2003; Casson & Hetherington, 2010; Lawson & Blatt, 2014; Matthews *et al*., 2018; Faralli *et al*., 2020). Stomatal density is known to vary within (Weyers & Lawson, 1997; Weyers *et al*., 1997) and between species (Ticha, 1982) and is also dependent on growth conditions (Woodward, 1987; Morison & Lawson, 2007; Stevens *et al*., 2021). The distribution of stomata can either be confined to one leaf surface – the abaxial surface (hypostomatous), or much less commonly, the adaxial surface (hyperstomatous) – or they can be present on both (amphistomatous; Parkhurst, 1978), which is the most conventional arrangement. Amphistomatous leaves can be further subdivided into dorsoventral or isobilateral species, where dorsoventrality is defined as having palisade mesophyll cells (which perform the majority of CO_2_ uptake) positioned nearest to the upper leaf epidermis, while isobilateral describes species with palisade mesophyll cells at both the upper and lower epidermis (Rudall, 1980; Brodribb *et al*., 2007; Drake *et al*., 2019). In general, amphistomatous species tend to have higher gas exchange capacity compared with hypostomatous species (Mott & O'Leary, 1984; Beerling & Kelly, 1996), which could be a result of shorter diffusion pathways, as well as differences in boundary layer conductance (Mott *et al*., 1982; de Boer *et al*., 2012; Drake *et al*., 2019; Xiong & Flexas, 2020).

Amphistomaty does not necessarily mean that similar SDs are found on both leaf surfaces (Willmer & Fricker, 1996; Taylor *et al*., 2012), with most species exhibiting greater SD on the abaxial (AB) compared with the adaxial (AD) leaf surface (Pemadasa, 1979; Mott *et al*., 1982; Willmer & Fricker, 1996). The exception is the Graminae family (Pemadasa, 1979), which often exhibit equal densities on the two surface, although SD tends to be higher on the AD surface in wheat. The typical lower distribution on the AD surface is associated with reducing water loss due to the higher evaporative demand from incoming solar radiation (Willmer & Fricker, 1996). The distribution between the two surfaces can be referred to as *R* = SD_adaxial_/(SD_adaxial_ + SD_abaxial_) (Muir, 2018), with hypostomatous leaves having a ratio of zero (*R* = 0) and amphistomatous leaves with equal distribution between surfaces having a ratio of 0.5 (*R* = 0.5) (Muir, 2015, 2018). Earlier studies suggested that AD stomata play a minor role in gaseous diffusion (Lu, 1989) as they tend to be fewer in number (Pemadasa, 1979) and/or exhibit reduced stomatal sensitivity (Lu, 1989; Lu *et al*., 1993; Goh *et al*., 1995). Differential stomatal responses to light intensity (Travis & Mansfield, 1981) and quality (Pemadasa, 1982) have been reported in several species including *Commelina communis* and *Vicia faba*, which are commonly used in stomatal studies. These differences have been attributed to modifications in the activity of the guard cell H^+^ proton pump (Goh *et al*., 1995), cytoplasmic Ca^2+^ concentrations (De Silva *et al*., 1986), starch and potassium concentrations (Pemadasa, 1979) and a divergent sensitivity of Ca^2+^ and abscisic acid (ABA) (Wang *et al*., 2008) between stomata on the two surfaces.

Stomatal behavior in isobilateral amphistomatous species (including Gramineae species, such as wheat), has been shown to operate independently, whereby stomata on AB and AD leaf surfaces can respond separately to external stimuli (Mott & Parkhurst, 1991; Richardson *et al*., 2017), such as differences in light intensity and CO_2_ concentration ([CO_2_]) (Long *et al*., 1989; Mott & Peak, 2018). Wang *et al*. (2008) suggested that the different stomatal responses of the two surfaces of the same leaf are due to differential light exposure, as the AD leaf surface is exposed to direct radiation, whilst the AB surface is shaded by itself, receiving light transmitted through the mesophyll and/or reflected from its surroundings (Vogelmann & Evans, 2002). Richardson *et al*. (2020) reported un‐coordinated stomatal responses on the two leaf surfaces in amphistomatous leaves that were driven by differences in leaf hydraulics, and this finding supports the idea of a tight coupling between stomatal behavior and leaf water supply (Flexas *et al*., 2014; McElwain *et al*., 2015). On the other hand, other studies have reported a coordinated response between the upper and lower leaf surfaces (Yera *et al*., 1986), although to date, the majority of these studies have not focused on key crop species. Wheat has more stomata on the AD surface; however, the functional relevance and benefits of this atypical anatomical characteristic are not well understood. To investigate the functional impact of wheat stomatal anatomy on gas exchange and photosynthesis, we developed a bespoke gas exchange chamber (split‐chamber; following earlier designs of Mott & O'Leary, 1984; Long *et al*., 1989) to separately determine the dynamic response of *A* and *g*_s_ simultaneously on both leaf surfaces. With this approach we addressed two key questions: firstly, do stomata on each leaf surface contribute equally to overall leaf gas exchange? And secondly, do stomata on each leaf surface operate independently, or are responses coordinated to some extent? We examined these responses in eight wheat cultivars (parents of the NIAB MAGIC population), and as a direct comparison to wheat, the dicot species *Phaseolus vulgaris* was included in the study. This crop exhibits a more ‘conventional’ stomatal distribution, in which the majority of stomata are found on the AB leaf surface.

## Materials and Methods

### Plant growth conditions

Eight varieties of *Triticum aestivum* L. (Alchemy, Brompton, Claire, Hereward, Rialto, Robigus, Soissons and Xi19; NIAB, Norwich, UK) were grown alongside one variety of *Phaseolus vulgaris* L. (Safari; Kings Seeds, Essex, UK). The wheat varieties included in this study were reported by Gardner *et al*. (2016) to represent 80% of the single nucleotide polymorphism variation in north‐west European bread wheat. Both plant species were germinated in a controlled environment cabinet (Adaptis, Conviron, Manitoba, Canada), with photosynthetically active photon flux density (PPFD) maintained at 200 ± 10 μmol m^−2^ s^−1^ at canopy height, under a 15 h : 9 h, light : dark photoperiod, at a constant temperature of 22°C and 1.1 kPa vapour pressure deficit (VPD). At 14 d post‐emergence, the wheat plants were vernalized for 10 wk at 4°C, with at PPFD of 75 μmol m^−2^ s^−1^, over a 10 h : 14 h, light : dark photoperiod. Plants were then transferred to a temperature‐controlled glasshouse at a day/night air temperature of 25/17°C ± 5°C. Lighting was supplemented by sodium vapour lamps (600 W; Hortilux Schrèder, Monster, the Netherlands) when external solar radiation fell below 500 μmol m^−2^ s^−1^ PPFD during a 10 h period. All plants were grown in individual 5 l pots containing peat‐based compost (Levington's F2S; ICL, Ipswich, UK). Plants were kept well‐watered, in addition to a once weekly nutrient supplement with Hoagland's solution.

All wheat measurements were taken from the flag leaf, at Zadok’s growth stage 49 (GS49, first awns/scurs visible) to GS59 (ear emergence complete) (Zadoks *et al*., 1974). *Phaseolus vulgaris* measurements were taken on the youngest fully expanded primary leaves from plants that were 4–5 wk old.

### Split‐chamber system design and construction

**Fig. 1:**
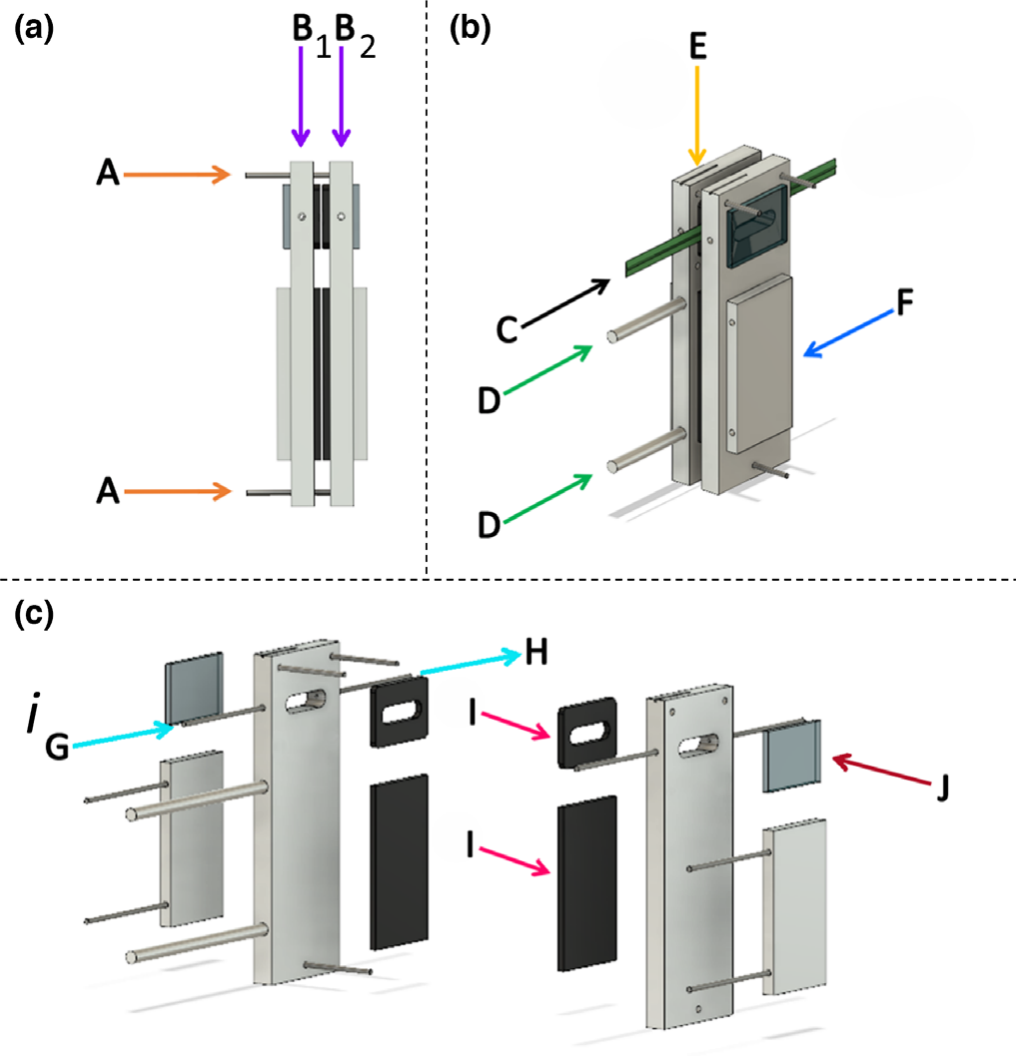
Bespoke split‐chamber cuvette for direct simultaneous and separate measurement of adaxial and abaxial leaf gas exchange. View from (a) side‐on and (b) front side with leaf clamped (labelled ‘C’), and (c) exploded computer‐aided design image of the chamber are shown. The cuvette was constructed from two aluminum plates (labelled ‘B_1_’ and ‘B_2_’) of equal dimensions: 150 mm (length) × 52 mm (width) × 10 mm (depth). Threaded rods (labelled ‘A’) between the two plates allowed fine tension adjustment to neoprene gaskets (labelled ‘I’), designed to prevent leaks, without damaging the sampled leaf. When the leaf (labelled ‘C’) is clamped between the chamber window (labelled ‘J*’*) it creates a tight seal, forming two separate compartments for gas exchange, with each chamber window area being 2.4 cm^2^. The air flow passes through the compartment, in the direction of ‘G’ to ‘H’. Integral water jackets (labelled ‘F’) provide temperature control via a recirculating water chiller, whilst the temperature is recorded by thermocouples measuring air and leaf temperature inserted into the chamber at the access point at the top (labelled ‘E’). Protruding rods (labelled ‘D’) allowed secure attachments of the chamber to the light source (not shown). [Colour figure can be viewed at wileyonlinelibrary.com]

A cuvette was specifically designed (University of Essex, Essex, UK) to simultaneously measure independent gas exchange from the AD and AB leaf surfaces. In brief, the cuvette was constructed from two water‐jacketed aluminum plates of equal dimensions – 150 mm (length) × 52 mm (width) × 10 mm (depth) (see Fig. 1) – which used a neoprene gasket to enclose and sample a projected leaf area of 2.4 cm^2^. Discrete gas flow to the AD and AB leaf surfaces was achieved by having separate gas flows to each side of the cuvette. An integral water jacket provided temperature control via a recirculating water chiller (BC20; Fisher Scientific, Loughborough, UK). Two cross‐calibrated Li‐6400 infrared gas analyzers (IRGAs; Li‐Cor Inc., Lincoln, NE, USA), a certified 1000 ppm CO_2_‐in‐air canister (BOC, Woking, UK) and a dew point generator (Li‐610; Li‐Cor) provided a known flow rate, [CO_2_] and [H_2_O] to each leaf surface. Additionally, this pair of Li‐6400 systems also measured the photosynthetic CO_2_ and H_2_O sample gas concentrations in the constructed cuvette used to calculate photosynthesis. Air and leaf temperatures were recorded using Type E thermocouples (Omega Engineering, Manchester, UK) independently for the AD and AB leaf surfaces. The thermocouples (shielded from incoming radiation using an aluminum foil hat) used to measure leaf temperature were 0.25 mm in diameter, and therefore the whole surface of the thermocouple was in contact with the leaf. Incident light at specific PPDF values was provided to each leaf surface by independently controlled white LED arrays (IsoLight 400; Technologica, Essex, UK; Supporting Information Fig. S1), which were calibrated using a quantum sensor (Li‐250A; Li‐Cor).

### Preliminary tests for the split‐chamber system

During gas analysis, flow rates to each leaf surface were continuously monitored by two cross‐calibrated in‐line flow sensors (FLR1005‐D; Omega Engineering) to ensure that they remained equal. These were positioned on the cuvette out‐flows, before gas analysis. Although this test confirmed the absence of leaks out of the cuvette, it was also necessary to check for flow transfer (or leaks) between the two sides of the cuvette. Therefore, preceding each set of measurements, the two sides of the cuvette were separated by a fully turgid leaf, and a temporary flow differential of 500 μmol air s^−1^ was applied between them. The in‐line flow meters (FLR1005‐D) were used to check that there was no leakage of flow between the sides of the cuvette by confirming that the aforementioned flow differential was still apparent at the outlets of the cuvette. Once the cuvette had been checked for leaks, a manometer (P200UL; Digitron, Port Talbot, UK) was used to confirm that the air pressure was equal on both sides of the cuvette. Any pressure differential would cause an ambiguity in the calculation of photosynthesis. Therefore, a gas‐impermeable insert was used to isolate each side of the cuvette, and the pressure was checked at the measurement flow rate of 500 μmol air s^−1^.

Diffusional leaks between the inside and outside of the chamber were quantified by precisely measuring the bulk airflow entering and leaving each surface of the leaf. Parity of these in/out flows for the two leaf surfaces confirmed that there were no detectable leaks. During the development of the chamber, this procedure of using flows to detect leaks from the chamber was validated by inserting gas‐impermeable material into the chamber and separating the two surfaces. Increasing the [CO_2_] from 400 to 1000 μmol mol^−1^ at both surfaces invoked a large diffusion gradient for CO_2_ between the inside and outside of the chamber, and no difference was observed between chamber input and output [CO_2_], confirming that there was no gas leakage from the chamber.

### Boundary layer conductance for the split‐chamber cuvette

Conventionally, gas exchange measurements are expressed in terms of ‘per unit projected leaf area’, and determining *g*_s_ requires knowledge of the boundary layer conductance *g*_b_, which is usually also determined on a projected leaf area basis. The leaf *g*_b_ was estimated separately for each surface using water‐saturated filter paper to simulate a leaf. If fully saturated, it can be assumed that the only resistance to transpiration is the boundary layer; therefore, the following equation from the instruction manual provided with the Li‐6400 system, derived from Ehleringer (1989), was used to calculate the one‐sided *g*_b_ (mol m^−2^ s^−1^):

(Eqn 1) $$g_{b}=\mathrm{EP}/\left[2\left(e_{s}\;–\;e_{a}\right)\right]$$

where *E* is the transpiration rate (mol m^−2^ s^−1^), *P* is the atmospheric pressure (kPa), *e*_*s*_ is the water vapour pressure (kPa) at the surface of the filter paper and *e*_*a*_ is the air kPa. Gas flow into the cuvette (500 μmol s^−1^), water jacket temperature (22°C) and light intensity (< 10 μmol m^−2^ s^−1^) were kept constant during the testing for the *g*_b_, and were monitored using the in‐line flow sensors, light meters, and thermocouple/data logger systems. Following this method, *g*_b_ was calculated as 0.582 mol m^−2^ s^−1^ for each individual leaf surface. Flow rate was kept constant for all experiments, and the calculated *g*_b_ value was therefore used in all subsequent calculations.

### Individual leaf surface responses of *A* and *g*_s_ to a step change in photosynthetically active photon flux density

The split‐chamber system was used obtain individual leaf surface gas exchange measurements in response to a single step increase in PPFD at the AD leaf surface. The incoming air, [CO_2_], leaf temperature and VPD were kept at 400 μmol mol^−1^, 22°C and 1 ± 0.2 kPa respectively. Steady‐state *A* and *g*_s_ (defined as < 2% change in rate over 5 min) values were measured every 30 s for 10 min at a PPFD of 100 μmol m^−2^ s^−1^, after which the PPFD was increased to 1000 μmol m^−2^ s^−1^ in a single step and recorded for a further 50 min. These measurements were repeated three times per plant. For the first set of measurements (for all plants), only the AD leaf surface was illuminated, initially with a PPFD of 100 μmol m^−2^ s^−1^ which was then increased to 1000 μmol m^−2^ s^−1^; for the second set of measurements, this process was repeated with illumination from the AB leaf surface. For the third set of measurements, both leaf surfaces of wheat plants were illuminated (i.e. AD and AB simultaneously), but the PPFD values were halved, to 50 and then 500 μmol m^−2^ s^−1^, to give a comparable total PPFD.

### The response of *A* and *g*_s_ to gaseous flux restriction from individual leaf surfaces

To assess the impact on *A* and *g*_s_ of restricting gas exchange to a single leaf surface, a thin coating of silicon grease was applied to the AB surface of wheat plants. Measurements of the responses to the step change in PPFD (made using the using the split‐chamber system, as described in the previous paragraph) were repeated for wheat cultivars for which the initial responses were most different: Xi19 and Brompton. In addition to the measurements recorded while illuminating only the AD surface, a second set of comparative measurements was made while illuminating both leaf surfaces. To ensure that the resultant incident PPFD was comparable between experiments, the PPFD values were halved when illuminating both the AD and AB surfaces (simultaneously) at 50 μmol m^−2^ s^−1^, and then at 500 μmol m^−2^ s. The greasing of the AB leaf surface caused an increase in temperature of *c*. 1.1°C and 0.6°C at 100 and 1000 μmol m^−2^ s^−1^ PPFD respectively.

### Response of *A* to changes in *C*_*i*_ of individual leaf surfaces

The response of *A* to changes in intercellular [CO_2_] (*C*_*i*_) of the AD and AB surfaces of wheat plants was measured simultaneously in the cultivars Brompton and Xi19 using the split‐chamber system. Photosynthesis was first stabilized at 400 μmol mol^−1^ and then decreased through the values 250, 150, 100, and 50 μmol mol^−1^. It was then returned to the initial value of 400 μmol mol^−1^ and increased through the values 550, 700, 900, 1100, 1300, and 1500 μmol mol^−1^. Photosynthesis was measured at each [CO_2_] value after *c*. 3 min. Saturating PPFD was kept at 1000 μmol m^−2^ s^−1^ for both the AD and AB leaf surfaces. Leaf temperature and VPD were 22°C and 1 ± 0.5 kPa respectively (the latter was maintained using a Li‐610 dew point generator; Li‐Cor).

### Response of *A* to changes in *C*_*i*_ and the impact of greasing leaf surfaces

The response of *A* to changes in *C*_*i*_ of the combined leaf surfaces in wheat cultivars Brompton and Xi19 was measured using a conventional IRGA (Li‐6400; Li‐Cor) with an integrated light source and standard 2 cm^2^ cuvette. Leaf temperature and VPD were maintained at 22°C ± 0.5°C and 1 kPa ± 0.3 kPa respectively. The *A*/*C*_*i*_ responses were repeated at PPFD values of 1000 and 2000 μmol m^−2^ s^−1^, with incident light always falling upon the AD leaf surface. The *A*/*C*_*i*_ protocol followed that for the split‐chamber measurements (as described in the preceding paragraphs). First, an *A*/*C*_*i*_ response was measured on an ungreased leaf as a control; on completion of this measurement, the AB surface was greased to inhibit gas exchange, and a second measurement was taken for the same area. Finally, a third *A*/*C*_*i*_ response was measured for an adjacent area of the leaf, in which the AD surface was greased.

### Measurements of stomatal density and size

Stomatal density was measured from leaf surface impressions taken using silicone impression material (Xantopren, Heraeus, Germany) following the methods described by Weyers *et al*. (1985). Six leaves per cultivar were measured at the middle of the leaf lamina. Stomatal density, GCL (used as a proxy for stomatal size) and pore length (PL) were then measured using a BX60 light microscope (Olympus, Essex, UK) set to a total magnification of ×100 for density measurements and ×400 for GCL and PL measurements.

Anatomical maximum stomatal conductance (*g*_smax_; mol m^−2^ s^−1^) was calculated from the measurements of density and stomatal dimensions (Eqn 2) following the equations of Franks & Farquhar (2001):

(Eqn 2) $$g_{\mathrm{smax}}=\left(d\cdot \mathrm{SD}\cdot a_{\max}\right)/\left(v\cdot \left(l+\left(\pi /2\right)\cdot \surd \left(a_{\max}/\pi \right)\right)\right)$$

where *d* is the diffusivity of water in air (m^2^ s^−1^, at 22°C) and *v* is the molar volume of air (m^3^ mol^−1^, at 22°C). Pore depth (*l*, μm) was equal to guard cell width at the centre of the stoma, represented as half the GCL. The mean maximum stomatal pore area (*a*_max_, μm^2^) was calculated assuming stomatal pores were elliptical, with the major axis equal to pore length and the minor axis equal to half pore length (see McElwain *et al*., 2015).

### Modelling gas exchange parameters

The maximum velocity of Rubisco for carboxylation (*V*_cmax_) and the maximum rate of electron transport demand for RuBP regeneration (*J*_max_) were calculated from the *A*/*C*_*i*_ response using equations from von Caemmerer & Farquhar (1981), and as described by Sharkey *et al*. (2007), using the Rubisco kinetic constants for wheat (Carmo‐Silva *et al*., 2010).

The response of *g*_s_ to the step change in PPFD was analysed following the method described by McAusland *et al*. (2016). In short, using the optimum function in R (www.r‐project.org; v.3.5.3), a model representing *g*_s_ as a function of time was fitted on each observed response:

(Eqn 3) $$g_{s}=\left(g_{\text{smax}}\;–\;r_{0}\right)\;e^{-e}\;\left(\frac{\lambda \;-\;\iota }{k}\;+1\right)+r_{0}$$

The model uses a sigmoidal equation with an initial time lag (the time before *g*_s_ starts to rise, *λ*, min), a time constant (the time to reach 63% of the variation, *k*, min), an initial value (*r*_0_, mol m^−2^ s^−1^) and a steady‐state target (the value when the plateau is reached, *g*_smax_, mol m^−2^ s^−1^). The time was set to 0 when PPFD was increased from 100 to 1000 μmol m^−2^ s^−1^.

### Statistical analysis

All statistical analysis was conducted in the R software environment (www.r‐project.org; v.3.5.3). For SD, GCL and *g*_smax_, the Shapiro–Wilk test was used to test for normality and Levene's test of homogeneity was used to determine if samples had equal variance. A log transformation was applied if the data were not normally distributed (*P* < 0.05, according to the Shapiro–Wilk test) to achieve normality and meet the modelling assumptions of an analysis of variance (ANOVA). Single‐factor differences were analysed using a one‐way ANOVA. When more than one factor existed, a two‐way ANOVA was applied with an interaction between the two factors. If a significant difference was found (*P* < 0.05) Tukey’s *post‐hoc* test was performed.

## Results

### Stomatal anatomy

**Fig. 2:**
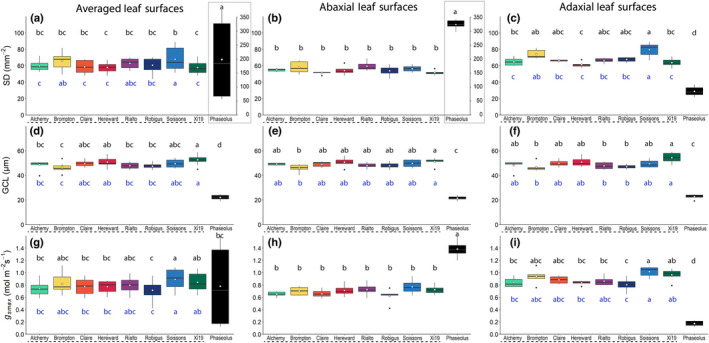
Boxplots of stomatal anatomy in eight wheat (*Triticum aestivum*) cultivars (underlined with a dashed line) and one French bean (*Phaseolus vulgaris)* cultivar. Variation (box and whisker plots displaying the distribution of biological replicates) and mean (white dot) values of stomatal density (SD; (a, b and c); notice the second *y*‐axis in the grey boxes for graphs (a and b)), guard cell length, representing stomatal size (GCL; (d, e and f)) and potential maximum anatomical stomatal conductance (*g*_smax_; (g, h and i), calculated from stomatal density and dimensions. Averaged abaxial and adaxial leaf surface measurements (a, d and g), abaxial leaf surface measurements (b, e and h) and adaxial leaf surface measurements (c, f and i) are shown. Black letters represent statistically significant differences (*P* < 0.05) between means of both species within the same graph, and blue letters represent statistically significant differences (*P* < 0.05) between means of wheat species only, using the results of a Tukey *post‐hoc* test following a two‐way analysis of variance (ANOVA; *n* = 6). [Colour figure can be viewed at wileyonlinelibrary.com]

Stomatal density over the leaf surface varied depending upon species and wheat cultivar (Fig. 2a–c). *Phaseolus vulgaris* had a significantly higher AB SD (+80%, *P* < 0.001) and lower AD SD (30% *P* < 0.001) than any of the wheat cultivars. Significant variation also exists between the wheat cultivars, with Soissons having a higher overall SD (*P* < 0.001) than all other wheat cultivars except for Brompton (+10%, *P* < 0.001) and Rialto. These differences in wheat SD were driven by significantly higher AD SD (Fig. 2c), as no significant differences in AB SD (Fig. 2b) were found between wheat cultivars. In general, *P. vulgaris* had significantly lower (*P* < 0.001) GCL than wheat on both leaf surfaces, and GCL in wheat varied between cultivars and leaf surfaces. For example, the cultivar Xi19 had a significantly larger GCL (+12%) than Brompton on both leaf surfaces (*P* < 0.05). Therefore, Brompton had one of the highest SD values, matched with the smallest GCL; by contrast, Xi19 exhibited the lowest SD value, with the largest GCL. On the AB surface, *P. vulgaris* had the highest SD value, which was correlated with smallest GCL value; however, the same was not true for the AD surface, which had the lowest SD value and a small GCL value.

Together, SD and stomatal size measurements were used to calculate *g*_smax_ for all measured plants, assuming stomata were fully open. Significant differences in leaf *g*_smax_ between species (*P* < 0.001) were observed (Fig. 2g–i)*. Phaseolus vulgaris* had a significantly higher *g*_smax_ value than wheat for the AB surface, but a lower value for the AD leaf surface (Fig. 2h,i; *P* < 0.001), following the differences in SD. Significant differences in leaf *g*_smax_ between wheat cultivars (*P* < 0.001) were apparent for the AD leaf surface but not for the AB leaf surface. This variation in *g*_smax_ at the leaf surface level was correlated with SD (*P* < 0.005) but not GCL, further supporting the idea that SD has an greater influence on *g*_smax_ than stomatal size (Lawson *et al*., 1998a,b). However, *g*_smax_ and SD did not follow identical trends: between cultivars, more significant differences in SD than *g*_smax_ were observed. Furthermore, there were no correlations between SD and *g*_smax_ at the individual leaf surface level, demonstrating that SD and GCL can counterbalance each other in terms of *g*_s_ contribution (as previously noted by Lawson & Morison, 2004; Harrison *et al*., 2020). This also provides an explanation as to why, for example, Brompton, Rialto and Xi19 had similar total *g*_smax_ values, despite their differing SD values (Fig. 2g).

### Gas exchange

#### Responses of *g*_s_ and *A* to a step change in photosynthetically active photon flux density

**Fig. 3:**
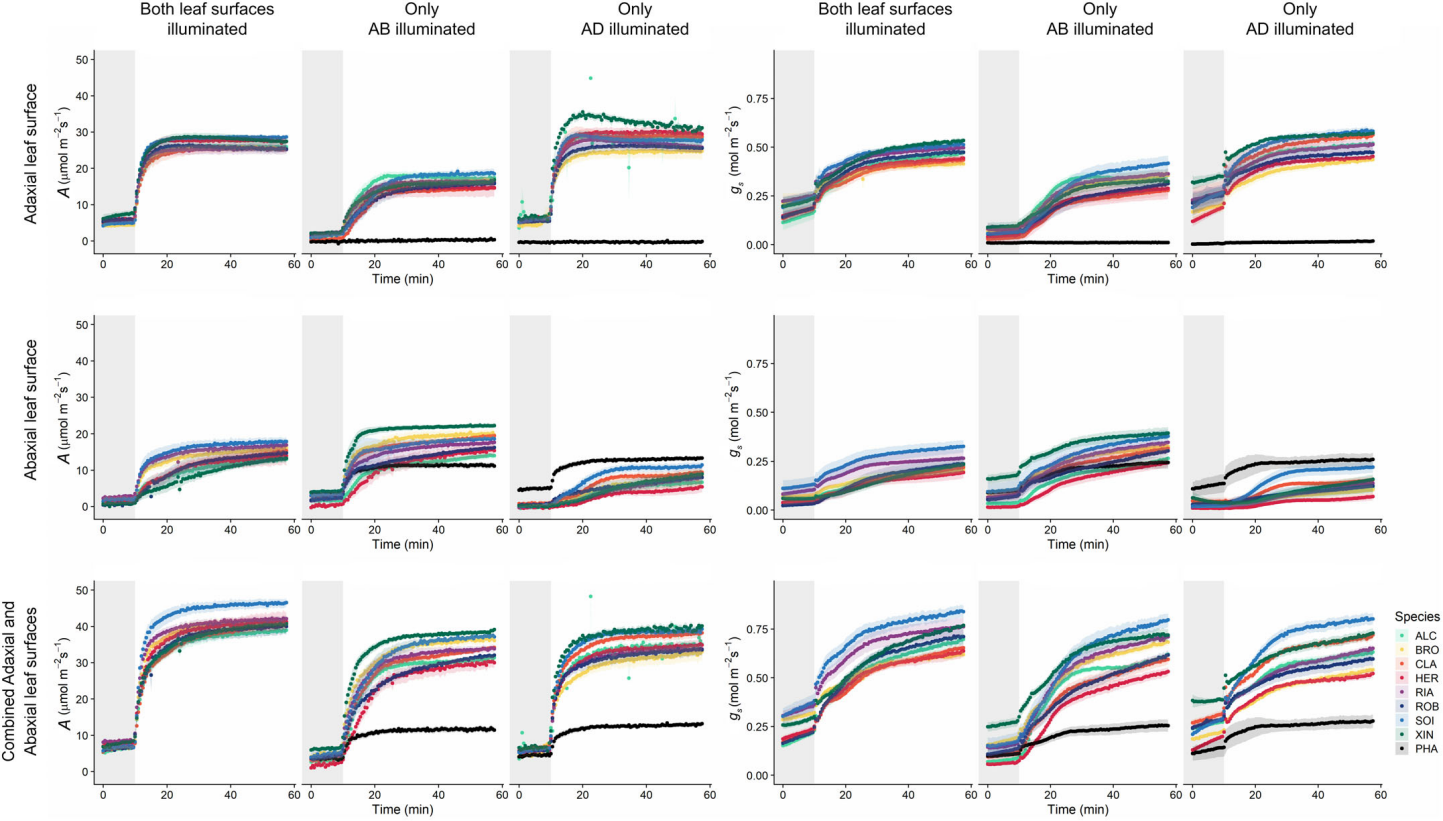
Response of stomatal conductance (*g*_s_; mol m^−2^ s^−1^) and net CO_2_ assimilation (*A*; μmol m^−2^ s^−1^) to a step increase in photosynthetically active photon flux density (PPFD) for French bean (PHA) and eight wheat cultivars – Alchemy (ALC), Brompton (BRO), Claire (CLA), Hereward (HER), Rialto (RIA), Robigus (ROB), Soissons (SOI) and Xi19 (XIN) – using the split‐chamber cuvette. Separate responses are shown for the adaxial and abaxial leaf surfaces, in addition to the combined response of both surfaces. Headings ‘Only AD illuminated’ and ‘Only AB illuminated’ represent the lighting regime, where plants were lit from the adaxial and abaxial leaf surfaces, respectively (100–1000 μmol m^−2^ s^−1^ PPFD), and ‘Both leaf surfaces illuminated’ represents plants lit from both sides (50–500 μmol m^−2^ s^−1^ PPFD). The grey box in each graph denotes low light conditions (50 or 100 μmol m^−2^ s^−1^ PPFD) with high light conditions (500 or 1000 μmol m^−2^ s^−1^ PPFD) introduced where the grey box ends. Gas exchange parameters (*A* and *g*_s_) were recorded at 30‐s intervals for 60 min; leaf temperature, [CO_2_] and leaf VPD were maintained at 22°C, 400 μmol mol^−1^ and 1 ± 0.2 kPa respectively. Error ribbons represent mean ± SE (*n* = 4–6). [Colour figure can be viewed at wileyonlinelibrary.com]

**Fig. 4:**
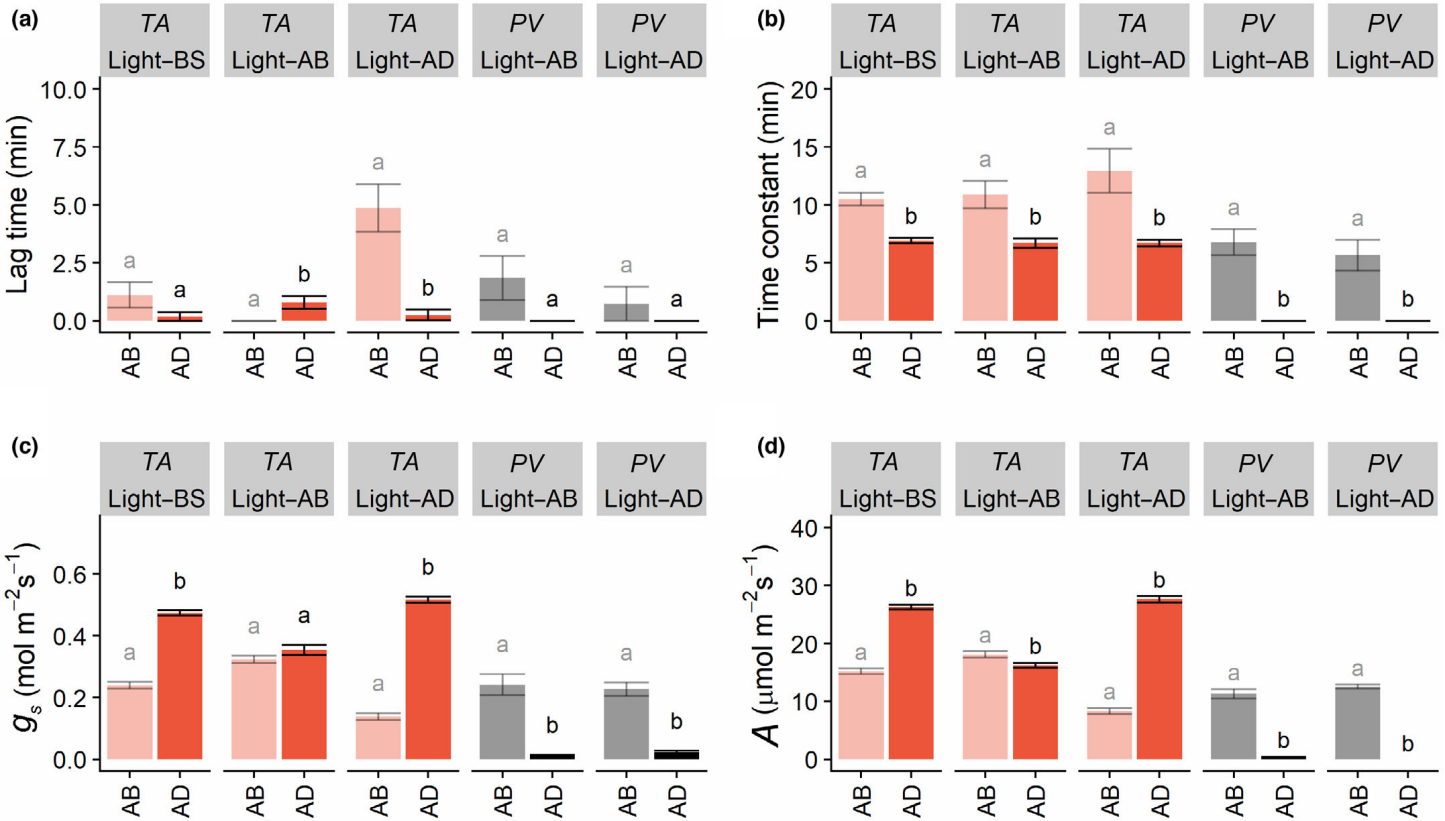
Variation in lag time in stomatal opening (a), the time constant for stomatal opening (b), stomatal conductance (c), and net CO_2_ assimilation (d), for the abaxial (AB) and adaxial (AD) leaf surfaces of *Phaseolus vulgaris* (*PV*, grey) and *Triticum aestivum* (*TA*, red) in response to a single step change in photosynthetically active photon flux density (PPFD). Headings ‘Light–AD’ and ‘Light–AB’ (in the grey boxes) represents the lighting regime, where the adaxial and abaxial leaf surfaces, respectively, were illuminated (100–1000 μmol m^−2^ s^−1^ PPFD); ‘Light–BS' represents plants lit from both sides (50–500 μmol m^−2^ s^−1^ PPFD). Different lowercase letters represent statistically significant differences (analysis of variance (ANOVA) and *post‐hoc* analysis; *P* < 0.05) between the means for each leaf surface of the light treatment (*n* = 4–6). [Colour figure can be viewed at wileyonlinelibrary.com]

To determine the impact of stomatal anatomical differences on leaf function, individual and combined leaf surface responses of *g*_s_ and *A* to a single step increase in PPFD (from 100 to 1000 μmol m^−2^ s^−1^) (Fig. 3) were examined. Both species and all cultivars exhibited a typical exponential‐type increase in *A* and *g*_s_ with increased PPFD (Fig. 3); however, there were some major differences between species and wheat cultivars depending on the surface measured and which side the leaf was illuminated from. After 50 min at high PPFD, *g*_s_ varied between the two species significantly (Fig. 4). *Phaseolus vulgaris* showed almost no *g*_s_ or *A* response from the AD side when illuminated from only the AD side or only the AB side, making these responses significantly different (*P* < 0.001) from all wheat species. The responses of *g*_s_ and *A* on the AB surface to increasing PPFD in *P. vulgaris* were similar, irrespective of whether illumination was received from the AB or AD leaf surface, and this was also true when both the AB and AD surfaces were illuminated simultaneously.

Within the eight wheat cultivars, there were more differences in *g*_s_ (Fig. 3) from individual and combined surfaces (depending on illumination, Fig. S2) than there were in *A* (Fig. S3). The highest *g*_s_ values were observed in cultivars Soissons and Xi19 (depending on surface and illumination) and these were significantly greater than the species with the lowest values, typically Hereward and Brompton (*P* < 0.05; Fig. S2). No significant differences in *A* were found in wheat, except in the AB leaf surface responses when illuminated from the AB leaf surface only (Fig. S3), where Xi19 had a significantly higher *A* than Hereward or Alchemy (*P* < 0.05). In wheat the greatest between‐cultivar differences in *g*_s_ were observed when the leaves were illuminated from both sides; however, both *A* and *g*_s_ were higher when the AD leaf surface was illuminated, compared with the AB leaf surface (Fig. 3). When only the AB surface was illuminated, *g*_s_ and *A* on the AB surface were strongly correlated (Figs S4–S6), but this was not the case for the AD surface, and this did not change with illumination. A visualization of the contribution from each surface in terms of *g*_s_, *A* and *C*_*i*_ during the step increase in PPFD was obtained by plotting the AD : AB ratios (Fig. S7). From these data it is clear, with a value > 1, that *g*_s_ from the AD surface always contributes more than *g*_s_ from the AB surface in all wheat cultivars, and this contribution was greatest when illumination was provided directly to the AD surface. The greater *g*_s_ AD : AB ratio also contributed to higher AD : AB values of *A*, although this was only apparent when illumination was provided to the AD surface or both surfaces, and the impact on *A* was greater earlier in the dynamic response, when slow *g*_s_ responses can limit the assimilation rate (Lawson *et al*., 2010). The AD : AB *C*_*i*_ ratio confirmed the initial *g*_s_ limitation of *A* over the time‐course of the experiment, with lower initial values which increased as *g*_s_ increased with PPFD. *Phaseolus vulgaris* showed completely different ratio kinetics, with extremely small AD : AB *g*_s_ values that were mirrored in the AD : AB *A* response, illustrating the fact that there was a limited contribution from the AD surface and that all gaseous exchange occurs through the AB surface, which was confirmed by the higher values of *C*_*i*_ in the AD : AB kinetics (Fig. S7).

Generally, greater differences were observed between wheat leaf surfaces compared with wheat cultivar‐specific differences; therefore, the subsequent experiments and results focused on the differences between surfaces, irrespective of wheat cultivars (a breakdown of all species and cultivars can be found in Fig. S8). An overview of the species‐specific responses of *A* and *g*_s_, separated by leaf surface and lighting treatment, is provided in Fig. 4. The temporal response of *g*_s_ was characterized by an initial delay (or lag time) before the exponential response (Fig. 4a), with no significant differences observed in *P. vulgaris* between surfaces. In wheat, no differences in the *g*_s_ lag time were observed when both surfaces were illuminated, however, the AB lag time was greatest when only the AD leaf surfaces were illuminated, and there was a significantly longer lag time at the AD surface when only the AB surface was illuminated. In order to assess the speed of stomatal responses, the time taken to reach 63% of the total *g*_s_ variation (or time constant) following the step increase of PPFD was assessed. In both species under all light treatments the *g*_s_ time constant was significantly lower on the AD surface compared to the AB surface under all light treatments (Fig. 4b). After 50 min of illumination (1000 μmol m^−2^ s^−1^ PPFD) of either the AD surface only or both leaf surfaces, *A* and *g*_s_ in wheat differed significantly (*P* < 0.05) between AB and AD leaf surfaces. Stomatal conductance was always higher at the AD leaf surface, achieving double that of the AB surface when illuminated from both sides or from the AD surface only (Fig. 4c). When only the AB surface was illuminated, the *A* and *g*_s_ contributions from both surfaces were nearly equal (Fig. 4c,d). In wheat, the pattern observed for *A* was similar to that observed for *g*_s_. *Phaseolus vulgaris* displayed higher values of *g*_s_ and *A* at the AB surface irrespective of which surface was illuminated (Fig. 4c,d).

#### Restricting vertical gas fluxes at the abaxial leaf surface

**Fig. 5:**
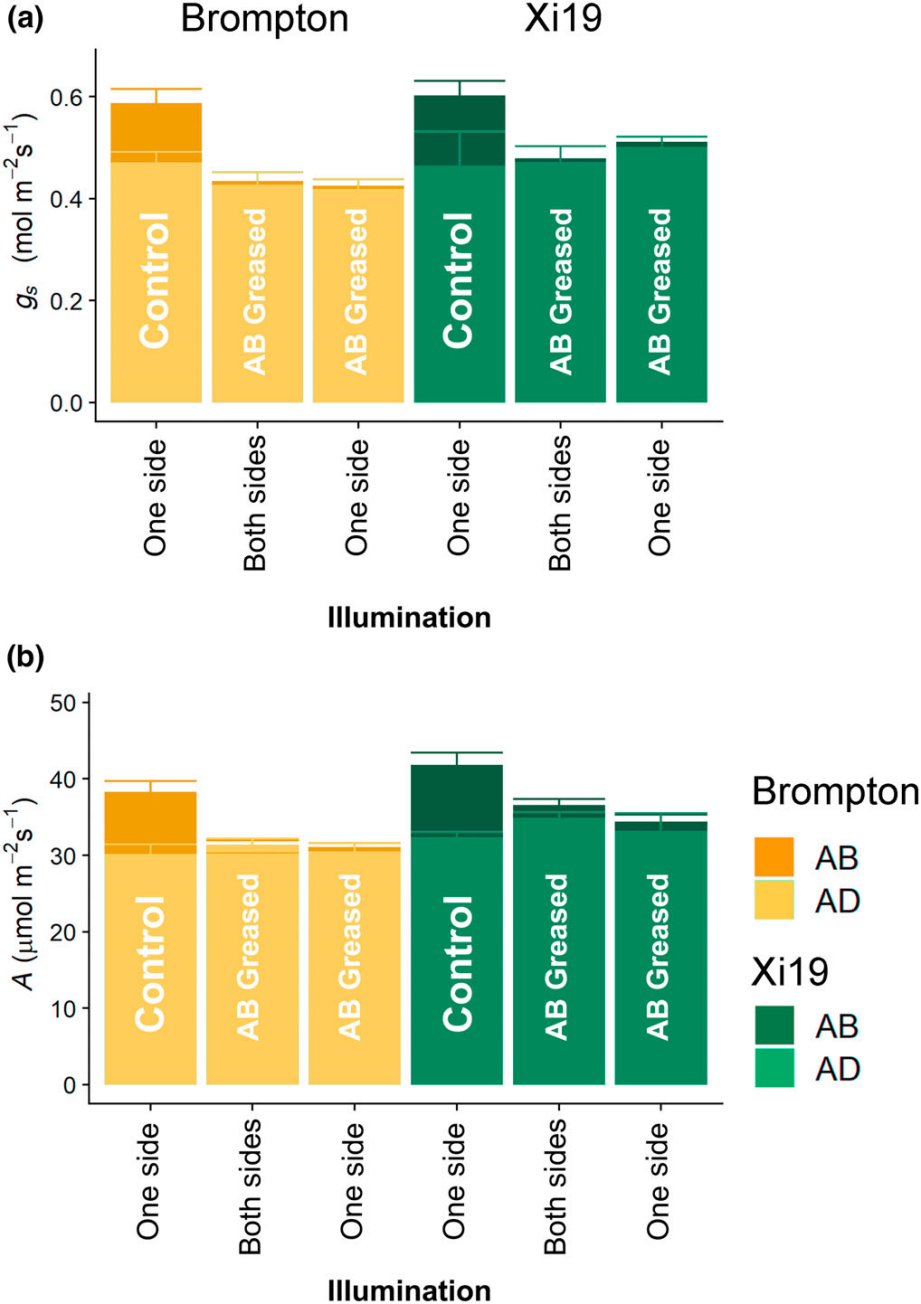
Stomatal conductance (*g*_s_) (a) and net CO_2_ assimilation (*A*) (b) for two selected wheat cultivars: Brompton and Xi19. Illumination was either incident on the adaxial (AD) surface only (‘One side’) at a photosynthetically active photon flux density (PPFD) of 100–1000 μmol m^−2^ s^−1^, or incident on both the adaxial and abaxial (AB) surfaces (‘Both sides’) at a PPFD of 50–500 μmol m^−2^ s^−1^ (per side). ‘Control’ bars represent an ungreased leaf, whereas ‘AB Greased’ denotes a leaf for which gas exchange was inhibited at the abaxial surface by a thin layer of silicon grease. Data are means calculated from the final five measurements taken after the step increase in PPFD, where *g*_s_ and *A* were considered steady state. Error bars represent mean 95% confidence intervals (*n* = 3–4). [Colour figure can be viewed at wileyonlinelibrary.com]

To further assess the diffusional contribution of the AB stomata to the overall *A* and *g*_s_, and to determine if AD stomatal behavior could compensate for any changes in AB *g*_s_, we examined gas exchange responses following a step increase in PPFD when vertical gaseous flux from the AB surface was prevented by blocking stomata with silicon grease (Fig. 5). Two wheat cultivars were selected (Brompton and Xi19) based on the observed differences in their stomatal anatomy (Fig. 2). When only the AD leaf surface was illuminated, the observed values of *A* and *g*_s_ were similar to those shown Fig. 3, and the contribution from the AD surface was greater (at *c*. 65% of total) than that from the AB leaf surface in both cultivars (Fig. 5). When vertical gaseous fluxes from the AB leaf surface were prevented, no significant changes or compensation were observed in the AD gas exchange in Xi19 or Brompton. When illumination was distributed evenly between the two surfaces (500 μmol m^−2^ s^−1^ on each surface; both sides) and the AB surface was greased, again no significant changes in AD *A* or *g*_s_ were observed compared with the AD control.

#### *A*/*C*_*i*_ gas exchange analysis of the adaxial and abaxial flag leaf surfaces

**Fig. 6:**
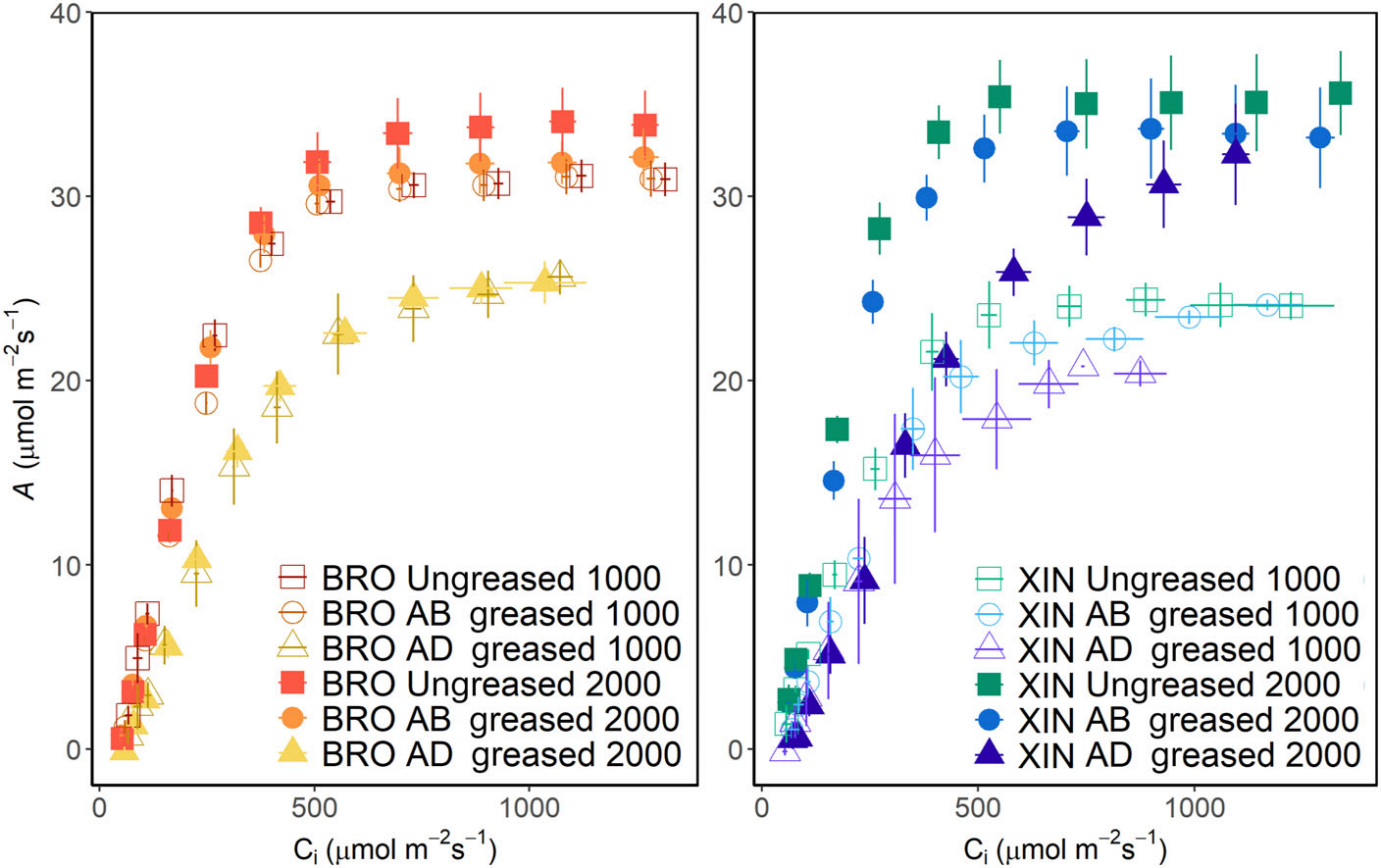
Wheat flag leaf response of net CO_2_ assimilation (*A*) to intercellular [CO_2_] (*C*_*i*_) between 50 and 1500 μmol m^−2^ s^−1^ using an infrared gas analyzer (IRGA) system. Measurements were taken at two photosynthetically active photon flux densities (PPFDs) (1000 and 2000 μmol m^−2^ s^−1^) for two wheat cultivars: Brompton and Xi19. To investigate the effect of inhibiting the gas exchange at the leaf surfaces, three sets of responses were measured: an ungreased leaf (‘Ungreased’), the abaxial surface greased (‘AB greased’) and the adaxial surface greased (‘AD greased’). Data represent means and SE (*n* = 4). [Colour figure can be viewed at wileyonlinelibrary.com]

**Fig. 7:**
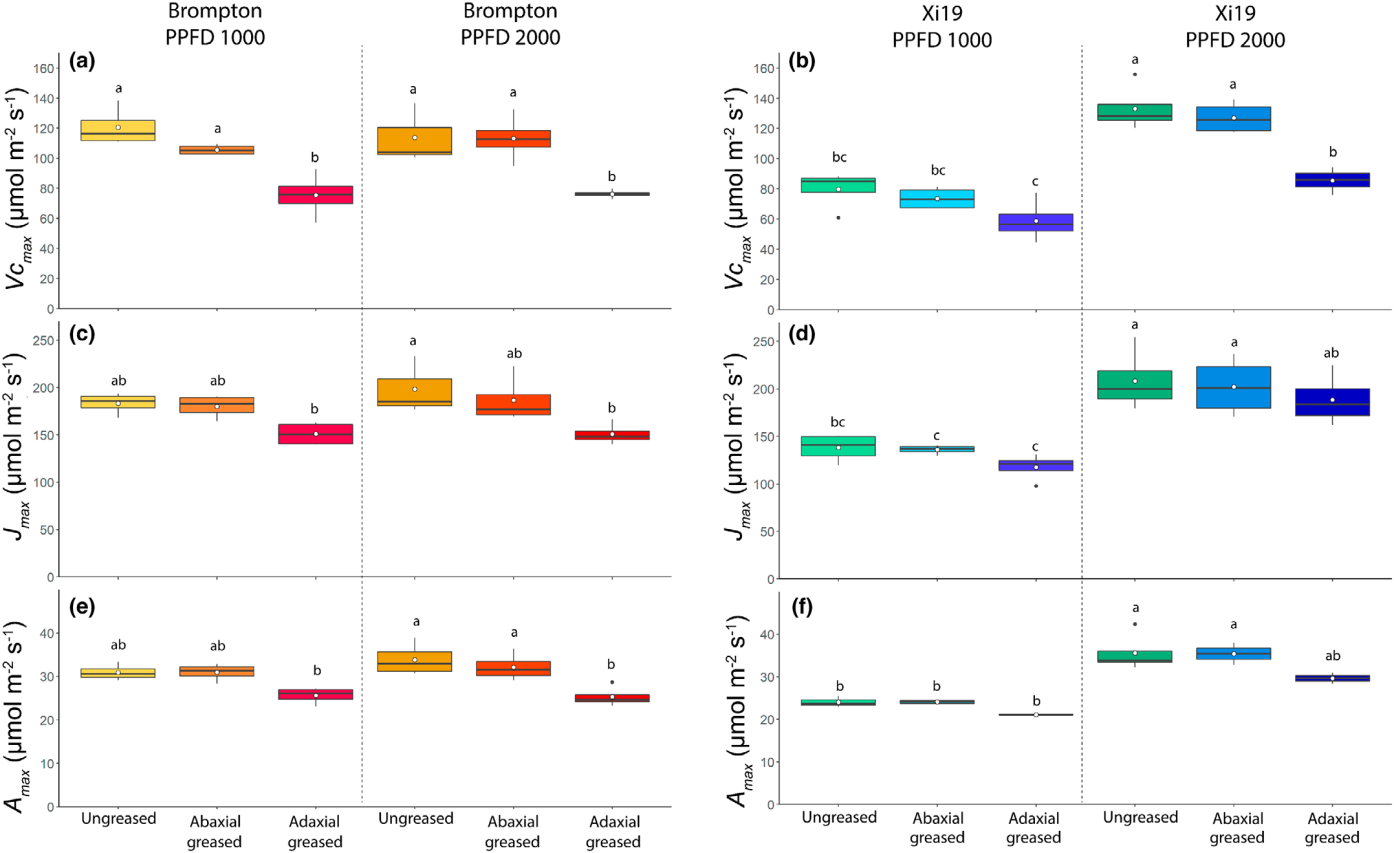
Boxplot showing variation and means (white dot) of the maximum rate of carboxylation (a, b) and maximum rate of electron transport (c, d) and the CO_2_‐saturated rate of photosynthesis (e, f) for flag leaves from the wheat cultivars Brompton and Xi19 measured at two photosynthetically active photon flux densities (PPFDs; 1000 and 2000 μmol m^−2^ s^−1^). To investigate the effect of inhibiting the gas exchange at the leaf surfaces, three sets of responses were measure: an ungreased leaf (‘Ungreased’), the abaxial surface greased (‘Abaxial greased’) and the adaxial surface greased (‘Adaxial greased’). Data represent means and SE (*n* = 4). Black letters represent statistically significant differences (*P* < 0.05) between means of both culitvars within the same graph, using the results of a Tukey *post‐hoc* test following a two‐way analysis of variance (ANOVA; *n* = 4). [Colour figure can be viewed at wileyonlinelibrary.com]

To determine the contribution from each surface to the overall leaf photosynthetic capacity, *A*/*C*_*i*_ curves were recorded for cultivars Brompton and Xi19 when either the AB or AD surfaces were greased, and the findings were compared with those recorded for ungreased leaves (Fig. 6). Response curves were recorded at two light levels, 1000 and 2000 μmol m^−2^ s^−1^, to assess the impact of transmitted light. In general, there were no differences between the two light levels for Brompton. However, for Xi19, doubling the PPFD from 1000 to 2000 μmol m^−2^ s^−1^ significantly increased *V*_cmax_, *J*_max_ and *A*_max_ (Fig. 7). Greasing the AB surface had no impact on rates of *A* (Fig. 6), *V*_cmax_, *J*_max_ or *A*_max_ at either light intensity (Fig. 7). It was only when the AD surface was greased that significant differences in photosynthetic capacity were observed. For example, Brompton showed significant decreases of 24%, 15% and 22%, respectively, in *V*_cmax_, *J*_max_ and *A*_max_ when measured at 2000 μmol m^−2^ s^−1^ PPFD (Fig. 7). A similar pattern was also observed in Xi19.

**Fig. 8:**
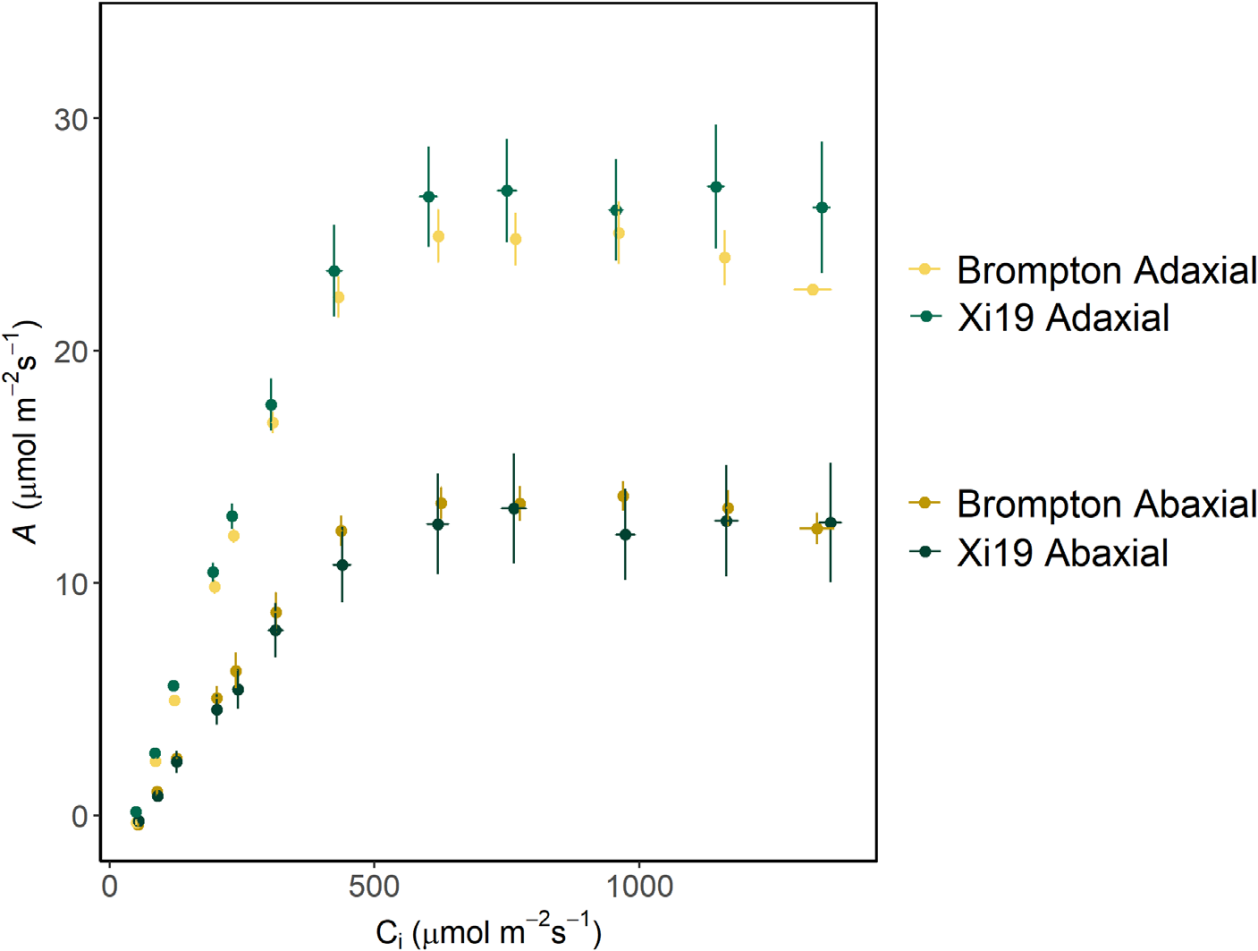
The response of net CO_2_ assimilation (*A*) to changing intercellular [CO_2_] (*C*_*i*_), from 50 and 1500 μmol m^−2^ s^−1^, for the adaxial and abaxial leaf surfaces using the split‐chamber system. Light intensity was 2000 μmol m^−2^ s^−1^ photosynthetically active photon flux density (PPFD), distributed evenly between the two surfaces (1000 μmol m^−2^ s^−1^) on each surface; total irradiance PPFD was 2000 μmol m^−2^ s^−1^ directed at both leaf surfaces for two selected wheat cultivars: Brompton and Xi19. Error bars represent mean ± SE (*n* = 4). [Colour figure can be viewed at wileyonlinelibrary.com]

**Fig. 9:**
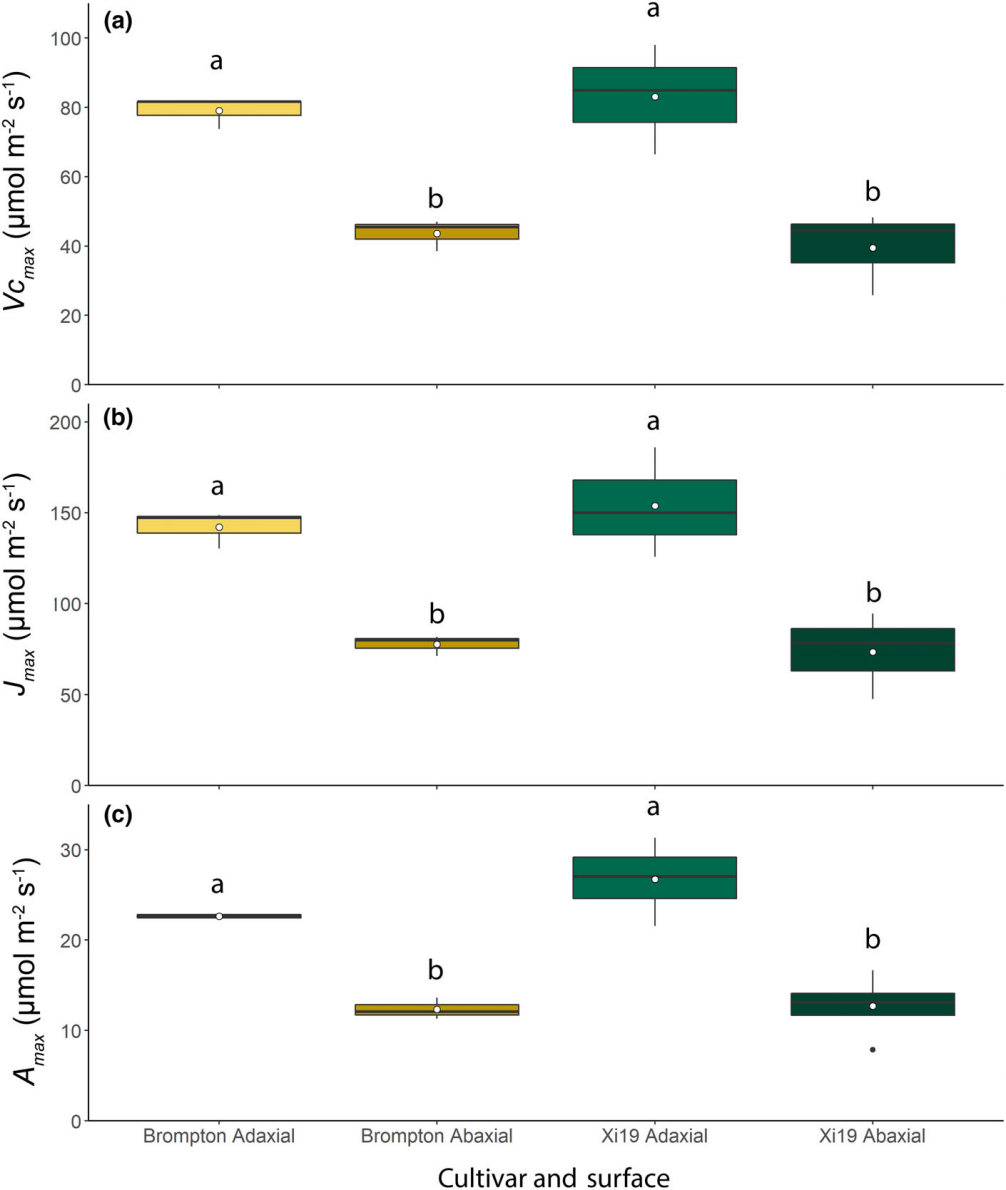
Boxplots showing variation and means (white dot) of the maximum rate of carboxylation (*V*_cmax_) (a) and maximum rate of electron transport (*J*_max_) (b) and the CO_2_‐saturated rate of photosynthesis (*A*_max_) (c) for the adaxial and abaxial surfaces using the split‐chamber cuvette for two wheat cultivars: Brompton and Xi19. Light intensity was 2000 μmol m^−2^ s^−1^ photosynthetically active photon flux density (PPFD), distributed equally between the two leaf surfaces. Error bars represent mean ± SE (*n* = 4). Different lowercase letters represent statistically significant differences (*P* < 0.05) between the means of each leaf surface and cultivar using the results of a Tukey *post‐hoc* test following a two‐way analysis of variance (ANOVA; *n* = 4). [Colour figure can be viewed at wileyonlinelibrary.com]

For the *A*/*C*_*i*_ response (Fig. 8), measured using the split‐chamber cuvette, when illuminating both sides of the leaves of Brompton and Xi19 cultivars, higher *A* was observed on the AD leaf surface, suggesting a difference in photosynthetic capacity between the two surfaces (Fig. 8). This was confirmed by the significantly higher values for *V*_cmax_, *J*_max_ and *A*_max_ on the AD surface compared to the AB surface for both Brompton and Xi19 (Fig. 9).

## Discussion

In Amphistomatous leaves, large differences in the numbers and distribution of stomata between AB and AD leaf surfaces are known (Ticha, 1982), although the resulting functional differences on overall leaf gas exchange are less well established (Mott *et al*., 1982; Xiong & Flexas, 2020; Santos *et al*., 2021) and there is no agreement on whether stomatal responses on both surfaces are synchronous (Mott *et al*., 1982; Yera *et al*., 1986). Amphistomaty has previously been associated with a greater capacity for gaseous diffusion through a reduction in the length of the CO_2_ diffusion pathway from the atmosphere to sites of carboxylation (Parkhurst, 1978, 1994; Franks & Beerling, 2009), contributing to an increased CO_2_ supply to the mesophyll (Parkhurst, 1978; Beerling & Kelly, 1996; Richardson *et al*., 2020) and high photosynthetic rates (Richardson *et al*., 2017), which is advantageous to plants in high light environments (Muir, 2019).

Here, we measure the simultaneous independent gas exchange of both leaf surfaces to examine how a greater SD on the AD leaf surface affects gas exchange in wheat, compared with a typical amphistomatous dicot (*P. vulg*aris) in which the majority of stomata are located on the AB surface. Significant differences in GCL and SD on the AB and AD surfaces that translated into differences in *g*_smax_ were observed between species (*P. vulgaris* and wheat) and within species (wheat). These differences in SD and GCL did not always translate into significant differences in the leaf *g*_s_ responses between the cultivars, suggesting an important role and a diversity of functional responses (Fig. 3). When we examined *g*_s_ responses to a step increase in illumination, there were no significant cultivar‐specific differences in the kinetics of these responses, and although some small differences in steady state *g*_s_ between wheat cultivars were observed, no differences in *A* were apparent (except for AB illumination; Fig. S3). However, what was immediately apparent from these findings was the large and generally greater contribution of AD leaf gas exchange in wheat, (which was not evident in *P. vulgaris*), in conjunction with more rapid responses (Figs 3, 4). These differences are linked to differences in photosynthetic capacity of the underlying mesophyll, as observed in the *A*/*C*_*i*_ analyses (Figs 8, 9), and the requirement for greater gaseous diffusion at the AD surface (Lawson & Blatt, 2014; Lawson & Matthews, 2020). High photosynthetic capacity in mesophyll cells associated with the AB (compared with the AD) leaf surface has been demonstrated in maize (Driscoll *et al*., 2006) and was correlated with a greater *SD* and higher *g*_s_.

The fact that no differences between AB *A* or AD *A* were observed (Figs 4, 5), irrespective of whether illumination was 500 or 1000 μmol m^−2^ s^−1^ PPFD indicates that mesophyll cells associated with each surface are light‐saturated at 500 μmol m^−2^ s^−1^ PPFD. These findings could imply that transmitted light from the opposite surface is of sufficient intensity to be as effective as higher intensity incident light for driving photosynthesis, and/or that the gas exchange is diffusionally constrained by *g*_s_. However, the latter seems to not be the case, as differences in *g*_s_ for the AD surface were dependent on illumination, but this did not influence *A*. These findings also imply that there is a limited contribution from AB *g*_s_ to the supply of CO_2_ for AD assimilation. This greatly supports the notion that photosynthetic capacity is lower in mesophyll cells associated with the AB leaf surface. Interestingly, *g*_s_ values were comparable when the AB leaf surfaces were illuminated, despite the differences in PPFD intensity at which stomatal opening typically occurs (Lawson *et al*., 2008). This could be explained by differences in stomatal sensitivity to both incident and transmitted light for the different leaf surfaces, as highlighted by Turner & Singh (1984) and Wang *et al*. (2008), although others have suggested no such differences in sensitivity (Yera *et al*., 1986). Alternatively, it could support the hypothesis that signals derived from the mesophyll determine stomatal responses (Lawson *et al*., 2018; Mott & Peak, 2018), which is further supported by the observations that AB and AD *g*_s_ (Fig. S5) are correlated, but only when the AD surface – which has the greater photosynthetic capacity, and therefore is mostly like the greatest contributor to any mesophyll signal – was illuminated.

Our findings suggest that there is some co‐ordination between the surfaces (Fig. 3), in that the kinetic *g*_s_ responses to a step change in PPFD were similar for the two surfaces, even though the absolute values achieved differed between the two surfaces (Fig. 4), suggesting that coordinated functional responses between stomata on the two surfaces are not driven by light alone, and that additional factors, including [CO_2_] and mesophyll driven signals, could play important roles (Yera *et al*., 1986; Lee & Bowling, 1992; Lawson *et al*., 2018; Mott & Peak, 2018). This is further supported by the *g*_s_ (AB : AD) ratio responses shown in Fig. S7, as, if coordination was driven entirely by light, this ratio would remain constant throughout the response, which was not the case when only the AD surface was illuminated. The idea of a mesophyll driven metabolite or signal that acts as a messenger initiating stomatal opening was first proposed by Dittrich & Raschke (1977) and supported by Wong *et al*. (1979). Since then, several studies have attempted to elucidate the signal (e.g., Lee & Bowling, 1992) and various suggestions have been put forward, including vapour ion signals (Mott *et al*., 2008; Sibbernsen & Mott, 2010), and sucrose concentration (Outlaw & Tarczynski, 1984; Outlaw & De Vlieghere‐He, 2001; Daloso *et al*., 2016), along with many others (Fujita *et al*., 2013; Lawson *et al*., 2014, 2018; Kottapalli *et al*., 2018). However, if a mesophyll signal alone was responsible for determining stomatal conductance, it would be expected that identical *g*_s_ values would be observed for both surfaces, or the AD : AB *g*_s_ ratio would correlate with AD : AB SD (Fig. S9), which was not the case here. Our findings do not support the proposal put forward by Yera *et al*. (1986) that coordinated stomatal responses between the two leaf surfaces are required to meet the photosynthetic requirements of the whole leaf.

To quantify the contribution each surface makes to gas exchange and photosynthesis, silicone grease was used to prevent vertical gaseous flux by blocking stomata on the AB leaf surface, and *g*_s_ and *A* were measured following a step increase in irradiance (Fig. 5). As no significant difference in AD *g*_s_ or *A* were observed between the greased (Fig. 5) and the nongreased treatments, it is apparent that AB gaseous fluxes contribute very little to the AD leaf surface *A*, that the AD stomata do not compensate for changes in AB *g*_s_, and that no overall leaf‐level *g*_s_ value is maintained. From these results, it appears most likely that the two surfaces act independently from one other. This does not agree with the findings of Mott & Peak (2018), who demonstrated that stomata on the AB surface opened further when gas exchange on the AD surface was blocked – this effect may be species‐specific.

The high rate of *A*, particularly for the AD surface, would suggest that the vertical profile of [CO_2_] through the leaf will be relatively high due to high consumption rates in the mesophyll layer close to the AD surface (Lawson & Morison, 2004; Morison & Lawson, 2007; Evans *et al*., 2009), which would limit CO_2_ supply from the AD surface to the AB surface and *vice versa*.

To evaluate potential vertical fluxes, we examined *A* when either of the surfaces was greased to prevent vertical CO_2_ fluxes through that surface. *A* was reduced, and this reduction was greater when the AD surface was greased, due to its higher photosynthetic capacity. The saturation of these curves at higher [CO_2_] indicates that each surface was both light‐ and CO_2_‐saturated, and that there was limited CO_2_ flux between the surfaces. The differences observed between the two cultivars also suggested that the diffusion limitation between the two leaf surfaces may be specific for each variety, representing a potential breeding target for the improvement of leaf photosynthesis.

To our knowledge, this is the first study that has measured the simultaneous but separate gas exchange of two leaf surfaces in real time in wheat and highlighted the difference in AD and AB *g*_s_ (despite only small SD differences). The high AD *g*_s_ values facilitate high *A*, as mesophyll cells associated with the AD surface have a high photosynthetic capacity. Furthermore, we have shown differences in stomatal kinetics between the two surfaces, with fast *g*_s_ responses on the AD surface, driven by both anatomical and biochemical components. The greater AD *g*_s_ will not only support higher *A* through increased CO_2_ diffusion, but could also be critical in more effective evapotranspiration for leaf cooling, to maintain optimal leaf temperatures for photosynthetic processes (i.e. for the light‐exposed flag leaf situated at the top of the canopy).

In conclusion, although SD in most amphistomatous species is typically higher on the AB leaf surface compared with the AD surface, the greater AD SD in wheat is necessary for supplying sufficient CO_2_ to support photosynthetic carbon assimilation in the underlying mesophyll. Having said this, AB gas exchange also makes a significant contribution (up to 50% depending on illumination) to whole‐leaf photosynthesis. Further studies are required to determine the roles of the differences in SD and behavior between the two surfaces, their significance in terms of evaporative cooling of the leaf, and whether these differences are consistent throughout the canopy. The findings also raise questions regarding the signals and mechanisms that control the differences in stomatal development between the two surfaces, particularly in relation to mesophyll photosynthetic capacity. Understanding a relationship such as this, and the underlying genetic controls, opens up new avenues of investigation and potentially unexploited targets via which stomatal development can be manipulated to generate plants with greater diffusional capacity and/or evaporative cooling that will be essential for future crop productivity.

## Author contributions

SW and TL designed the experiments and wrote the manuscript. SW executed all the experiments and acquired all the data, except for the greased *A*/*C*_*i*_ curves, which were completed by PD. SW, SV‐C and TL analysed data. SV‐C modelled and analysed induction data. SV‐C, JC, PD, AG and JVR contributed to the editing of the manuscript.

## Supporting information


**Fig. S1** Spectrum of white actinic LED light source used with the split‐chamber system, measured using a spectroradiometer.
**Fig. S2** Statistics for end responses of stomatal conductance to a step increase in photosynthetically active photon flux density for eight wheat cultivars – Alchemy, Brompton, Claire, Hereward, Rialto, Robigus, Soissons and Xi19 using the split‐chamber cuvette.
**Fig. S3** Statistics for end responses of net CO_2_ assimilation to a step increase in photosynthetically active photon flux density for eight wheat cultivars – Alchemy, Brompton, Claire, Hereward, Rialto, Robigus, Soissons and Xi19 – using the split‐chamber cuvette.
**Fig. S4** Correlation between leaf gas exchange and leaf anatomy parameters for a leaf with an illuminated abaxial surface.
**Fig. S5** Correlation between leaf gas exchange and leaf anatomy parameters for a leaf with an illuminated adaxial surface.
**Fig. S6** Correlation between leaf gas exchange and leaf anatomy parameters for a leaf illuminated from both sides.
**Fig. S7** Ratio of adaxial : abaxial values for stomatal conductance, net CO_2_ assimilation and intercellular CO_2_ concentration in response to a step increase in photosynthetically active photon flux density for eight wheat cultivars – Alchemy, Brompton, Claire, Hereward, Rialto, Robigus, Soissons and Xi19 – using the split‐chamber cuvette.
**Fig. S8** Variation in lag time in stomatal opening, the time constant for stomatal opening, steady state stomatal conductance, and net CO_2_ assimilation for the abaxial and adaxial leaf surfaces of *Phaseolus vulgaris* and eight wheat cultivars in response to a single step change in photosynthetically active photon flux density.
**Fig. S9** Boxplots of stomatal anatomy ratios for abaxial and adaxial leaf surfaces in eight wheat (*Triticum aestivum*) cultivars and one French bean (*Phaseolus vulgaris*) cultivar.
Please note: Wiley Blackwell are not responsible for the content or functionality of any Supporting Information supplied by the authors. Any queries (other than missing material) should be directed to the *New Phytologist* Central Office.

## Data Availability

The data that support the findings of this study are available from the corresponding author upon reasonable request.
