# Supplementary material for: Stomata on the abaxial and adaxial leaf surfaces contribute differently to leaf gas exchange and photosynthesis in wheat
Source: New Phytol. 2022 Jun 30;235(5):1743–56. doi: 10.1111/nph.18257 (PMC9545378; doi:10.1111/nph.18257)
Supplement: Supplementary file 1 — Fig. S1 Spectrum of white actinic LED light source used with the split‐chamber system, measured using a spectroradiometer. Fig. S2 Statistics for end responses of stomatal conductance to a step increase in photosynthetically active photon flux density for eight wheat cultivars – Alchemy, Brompton, Claire, Hereward, Rialto, Robigus, Soissons and Xi19 using the split‐chamber cuvette. Fig. S3 Statistics for end responses of net CO2 assimilation to a step increase in photosynthetically active photon flux density for eight wheat cultivars – Alchemy, Brompton, Claire, Hereward, Rialto, Robigus, Soissons and Xi19 – using the split‐chamber cuvette. Fig. S4 Correlation between leaf gas exchange and leaf anatomy parameters for a leaf with an illuminated abaxial surface. Fig. S5 Correlation between leaf gas exchange and leaf anatomy parameters for a leaf with an illuminated adaxial surface. Fig. S6 Correlation between leaf gas exchange and leaf anatomy parameters for a leaf illuminated from both sides. Fig. S7 Ratio of adaxial : abaxial values for stomatal conductance, net CO2 assimilation and intercellular CO2 concentration in response to a step increase in photosynthetically active photon flux density for eight wheat cultivars – Alchemy, Brompton, Claire, Hereward, Rialto, Robigus, Soissons and Xi19 – using the split‐chamber cuvette. Fig. S8 Variation in lag time in stomatal opening, the time constant for stomatal opening, steady state stomatal conductance, and net CO2 assimilation for the abaxial and adaxial leaf surfaces of Phaseolus vulgaris and eight wheat cultivars in response to a single step change in photosynthetically active photon flux density. Fig. S9 Boxplots of stomatal anatomy ratios for abaxial and adaxial leaf surfaces in eight wheat (Triticum aestivum) cultivars and one French bean (Phaseolus vulgaris) cultivar. Please note: Wiley Blackwell are not responsible for the content or functionality of any Supporting Information supplied by the authors. Any queries [file NPH-235-1743-s001.pdf]

New Phytologist Supporting Information Article title: **Stomata on the abaxial and adaxial leaf surface contribute differently to leaf gas exchange and photosynthesis in wheat**

Authors: **Shellie Wall[1], Silvere Vialet-Chabrand[1]** (orchid ID 0000-0002-2105-2825), **Phillip Davey[1], Jeroen Van Rie[2],** (orchid ID 0000-0002-5986-962X), **Alexander Galle[2],** (orchid ID 0000-0001-6927-7911), **James Cockram[3]** (orchid ID 0000-0002-1014-6463) **and Tracy Lawson[1*]**(orcid ID 0000-0002-4073-7221).

Article acceptance date: 6th April 2022

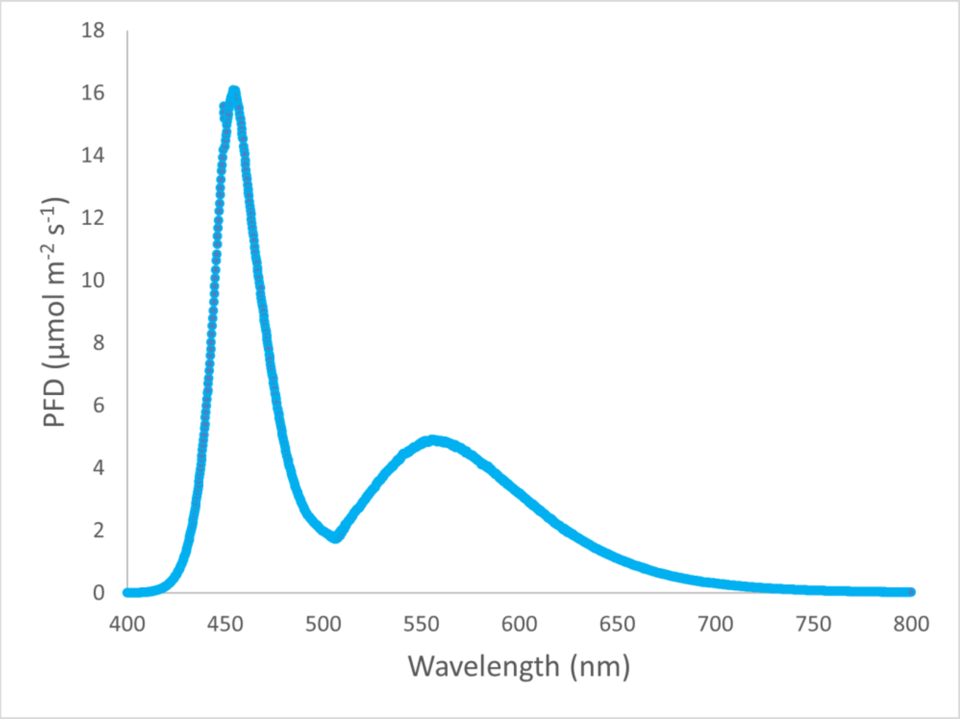

Fig. S1: Spectrum of white actinic LED light source (Iso light, Technologica) used with the split chamber, measured by a spectroradiometer (Spectrapen, PSI Systems, Czech republic).

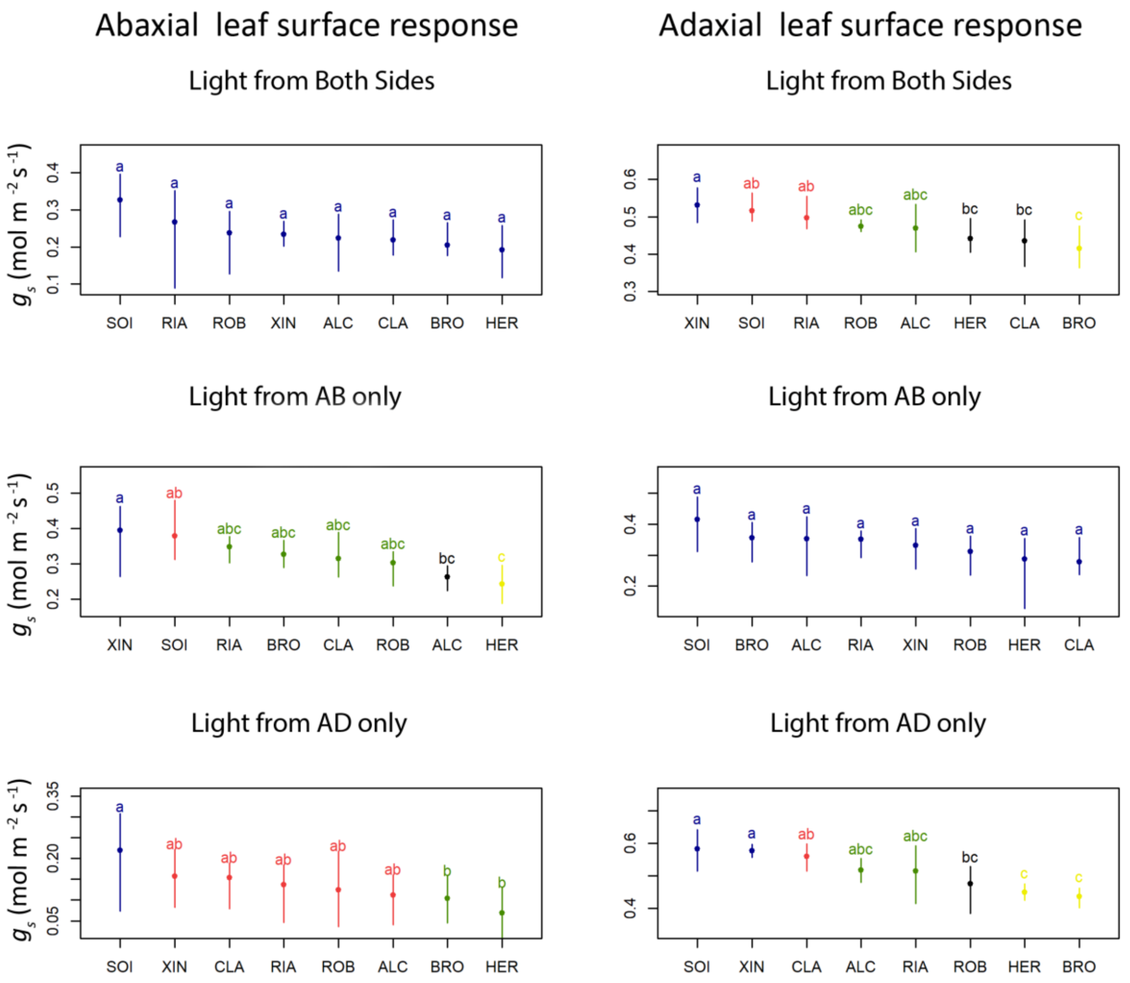

Fig. S2: Statistics for end responses of stomatal conductance (g_s; mol m^{-2} s^{-1}) to a step increase in PPFD for eight wheat cultivars, Alchemy (ALC), Brompton (BRO), Claire (CLA), Hereward (HER), Rialto (RIA), Robigus (ROB), Soissons (SOI) and Xi19 (XIN) using the split-chamber cuvette. Separate responses are shown for the adaxial and abaxial leaf surfaces, in addition to the combined response of both surfaces. Headings 'Light from AD only' and 'Light from AB only' represent the lighting regime, where plants were lit from the adaxial and abaxial leaf surface respectively (1000 μmol m^{-2} s^{-1} PPFD), and 'Light from Both sides' represents plants lit from both sides (500 μmol m^{-2} s^{-1} PPFD). Gas exchange parameters were recorded at 60 min, leaf temperature, [CO_2] and leaf VPD were maintained at 22 °C, 400 μmol mol^{-1} and 1 ± 0.2 kPa respectively. Different letters represent statistically significant differences ($p < 0.05$) between means of each leaf surface of the light treatment ($n = 4$-6).

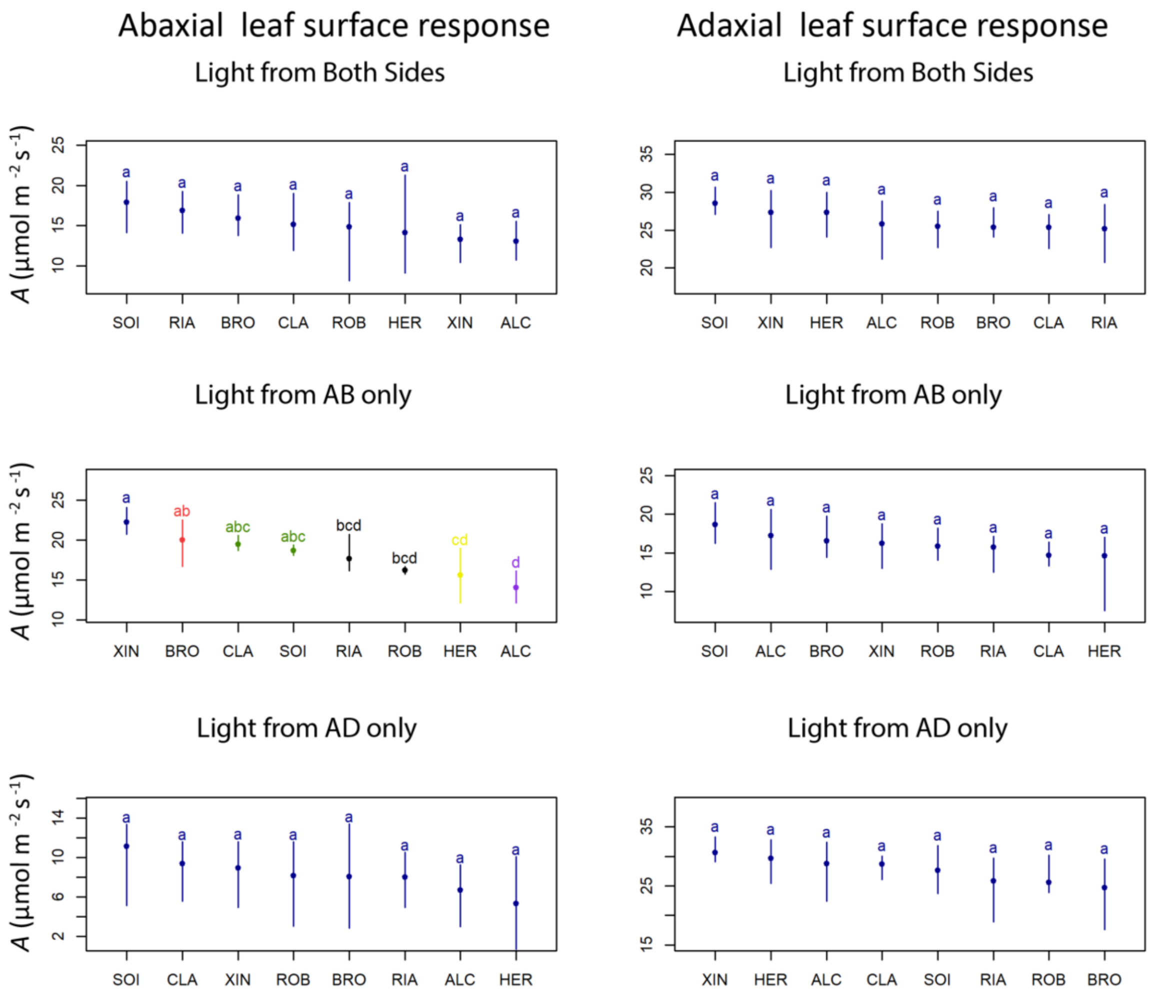

Fig. S3: Statistics for end responses of net CO_2 assimilation (A; µmol m^{-2} s^{-1}) to a step increase in PPFD for eight wheat cultivars, Alchemy (ALC), Brompton (BRO), Claire (CLA), Hereward (HER), Rialto (RIA), Robigus (ROB), Soissons (SOI) and Xi19 (XIN) using the split-chamber cuvette. Separate responses are shown for the adaxial and abaxial leaf surfaces, in addition to the combined response of both surfaces. Headings 'Light from AD only' and 'Light from AB only' represent the lighting regime, where plants were lit from the adaxial and abaxial leaf surface respectively (1000 µmol m^{-2} s^{-1} PPFD), and 'Light from Both sides' represents plants lit from both sides (500 µmol m^{-2} s^{-1} PPFD). Gas exchange parameters were recorded at 60 min, leaf temperature, [CO_2] and leaf VPD were maintained at 22 °C, 400 µmol mol^{-1} and 1 ± 0.2 kPa respectively. Different letters represent statistically significant differences ($p < 0.05$) between means of each leaf surface of the light treatment (n = 4-6).

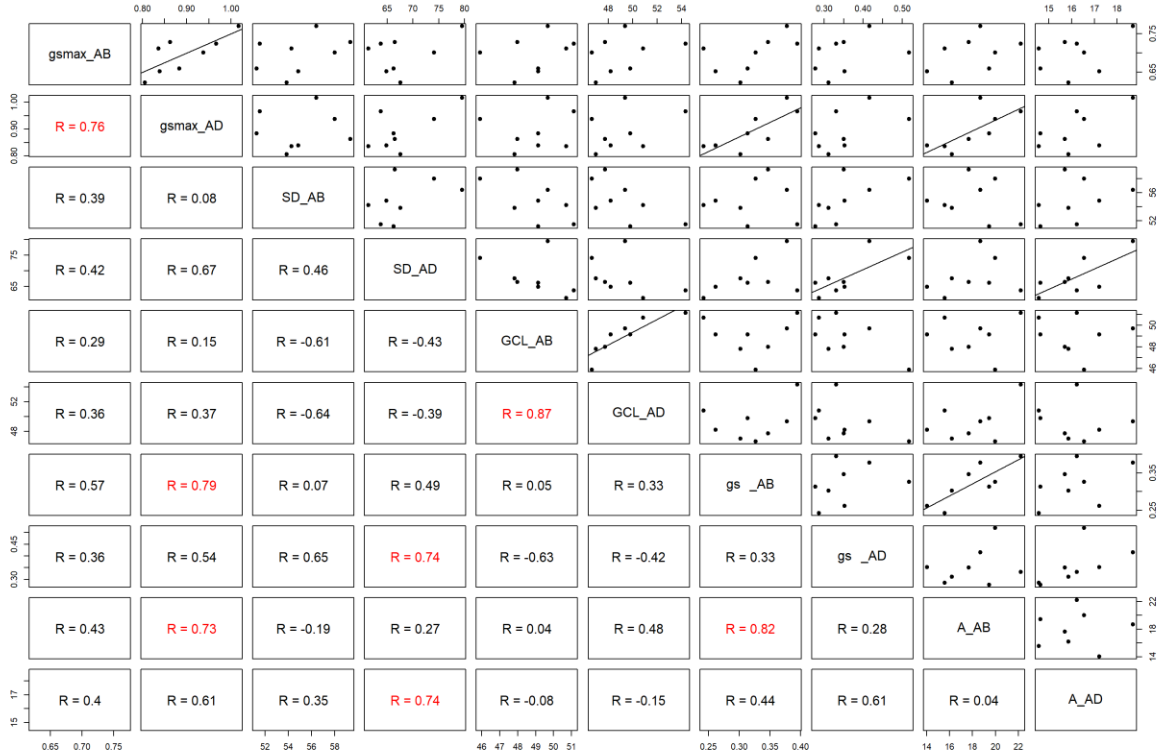

Fig. S4: Correlation between leaf gas exchange and leaf anatomy parameters from a leaf lit from the Abaxial leaf surface. Parameters with suffixes “AB” and “AD” represent the values for the abaxial and adaxial leaf surface respectively. gsmax: maximum stomatal conductance, SD; stomatal density, GCL; guard cell length, g_s; steady state stomatal conductance under 1000 µmol m^{-2} s^{-1}, A: steady state net CO_2 assimilation under 1000 µmol m^{-2} s^{-1}. R values for significant correlations are highlighted in red.

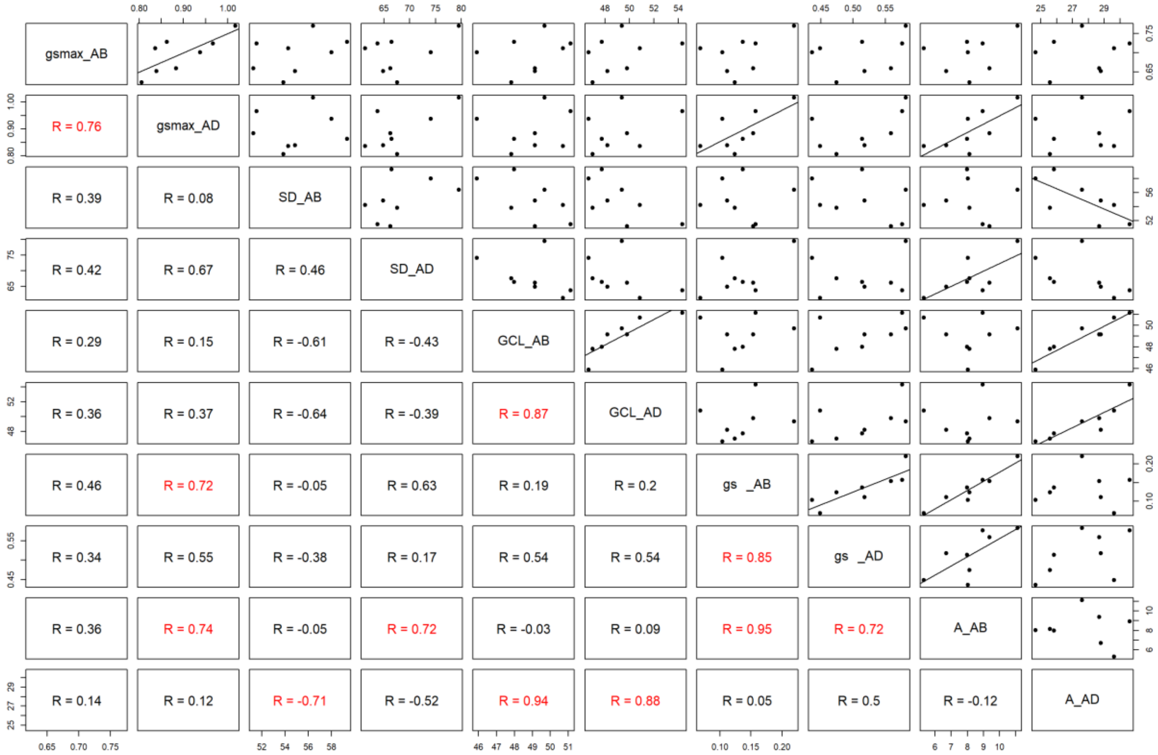

Fig. S5: Correlation between leaf gas exchange and leaf anatomy parameters from a leaf lit from the Adaxial side. Parameters with suffixes "AB" and "AD" represent the values for the abaxial and adaxial leaf surface respectively. g_{smax}: maximum stomatal conductance, SD; stomatal density, GCL; guard cell length, g_s; steady state stomatal conductance under 1000 µmol m^{-2} s^{-1}, A: steady state net CO_2 assimilation under 1000 µmol m^{-2} s^{-1}. R values for significant correlations are highlighted in red.

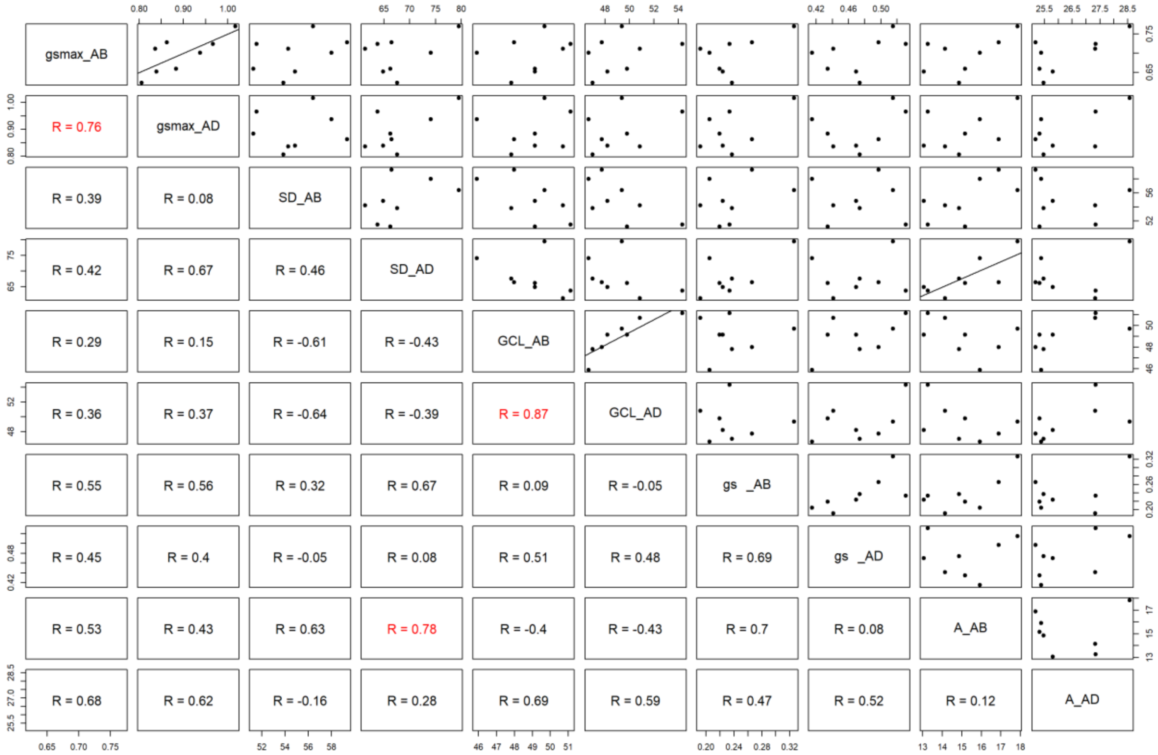

Fig. S6: Correlation between leaf gas exchange and leaf anatomy parameters from a leaf lit from both sides. Parameters with suffixes “AB” and “AD” represent the values for the abaxial and adaxial leaf surface respectively. g_{smax}: maximum stomatal conductance, SD; stomatal density, GCL; guard cell length, g_s; steady state stomatal conductance under 1000 µmol m^{-2} s^{-1}, A: steady state net CO_2 assimilation under 1000 µmol m^{-2} s^{-1}. R values for significant correlations are highlighted in red.

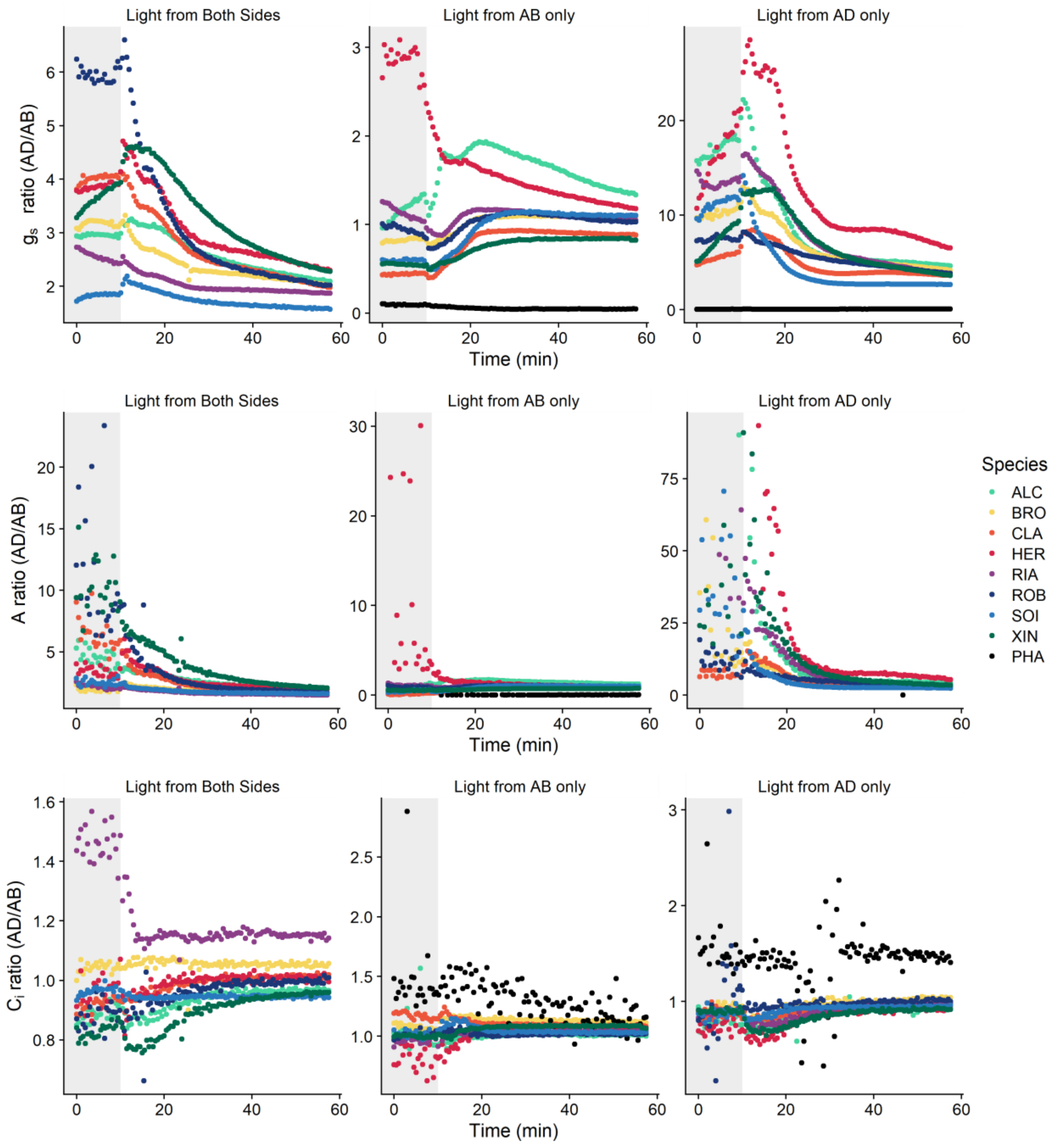

Fig. S7: Ratio of adaxial to abaxial values for stomatal conductance (g_s), net CO_2 assimilation (A) and intercellular CO_2 concentration (C_i) in response to a step increase in PPFD for eight wheat cultivars, Alchemy (ALC), Brompton (BRO), Claire (CLA), Hereward (HER), Rialto (RIA), Robigus (ROB), Soissons (SOI) and Xi19 (XIN) using the split chamber cuvette. Headings 'Light from AD only' and 'Light from AB only' represent the lighting regime, where plants were lit from the adaxial and abaxial leaf surface respectively (1000 µmol m^{-2} s^{-1} PPFD), and 'Light from Both sides' represents plants lit from both sides (500 µmol m^{-2} s^{-1} PPFD). Gas exchange parameters were recorded at 60 min, leaf temperature, [CO2] and leaf VPD were maintained at 22 °C, 400 µmol $mol^{-1}$and 1 ± 0.2 kPa respectively.

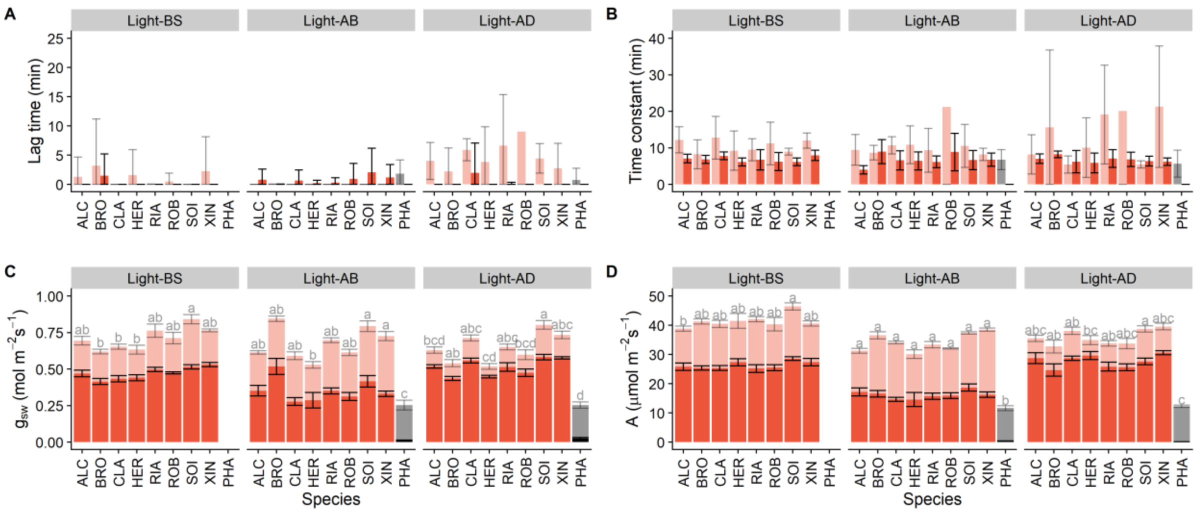

Fig. S8: Variation of lag time in stomatal opening (A), time constant for stomatal opening (B), steady state stomatal conductance (C), net CO_2 assimilation (D) for the abaxial (AB) and adaxial (AD) leaf surfaces of *Phaseolus Vulgaris* (P.V, grey) and eight wheat cultivars (Alchemy, Brompton, Claire, Hereward, Rialto, Robigus, Soissons and Xi19) to a single step change in PPFD. Headings 'Light-AD' and 'Light-AB' (grey banner) represents the lighting regime, where plants were lit from the adaxial and abaxial leaf surface respectively (100 – 1000 µmol m^{-2} s^{-1} PPFD) and 'Light-BS' (grey banner) represents plants lit from both sides (50 – 500 µmol m^{-2} s^{-1} PPFD). Error bars in (A) and (B) represent 95% confidence intervals and in (C) and (D) the standard error of mean. Different letters represent statistically significant differences ($p < 0.05$, Tukey HSD) between means of the added value for both leaf surfaces (n = 4-6).

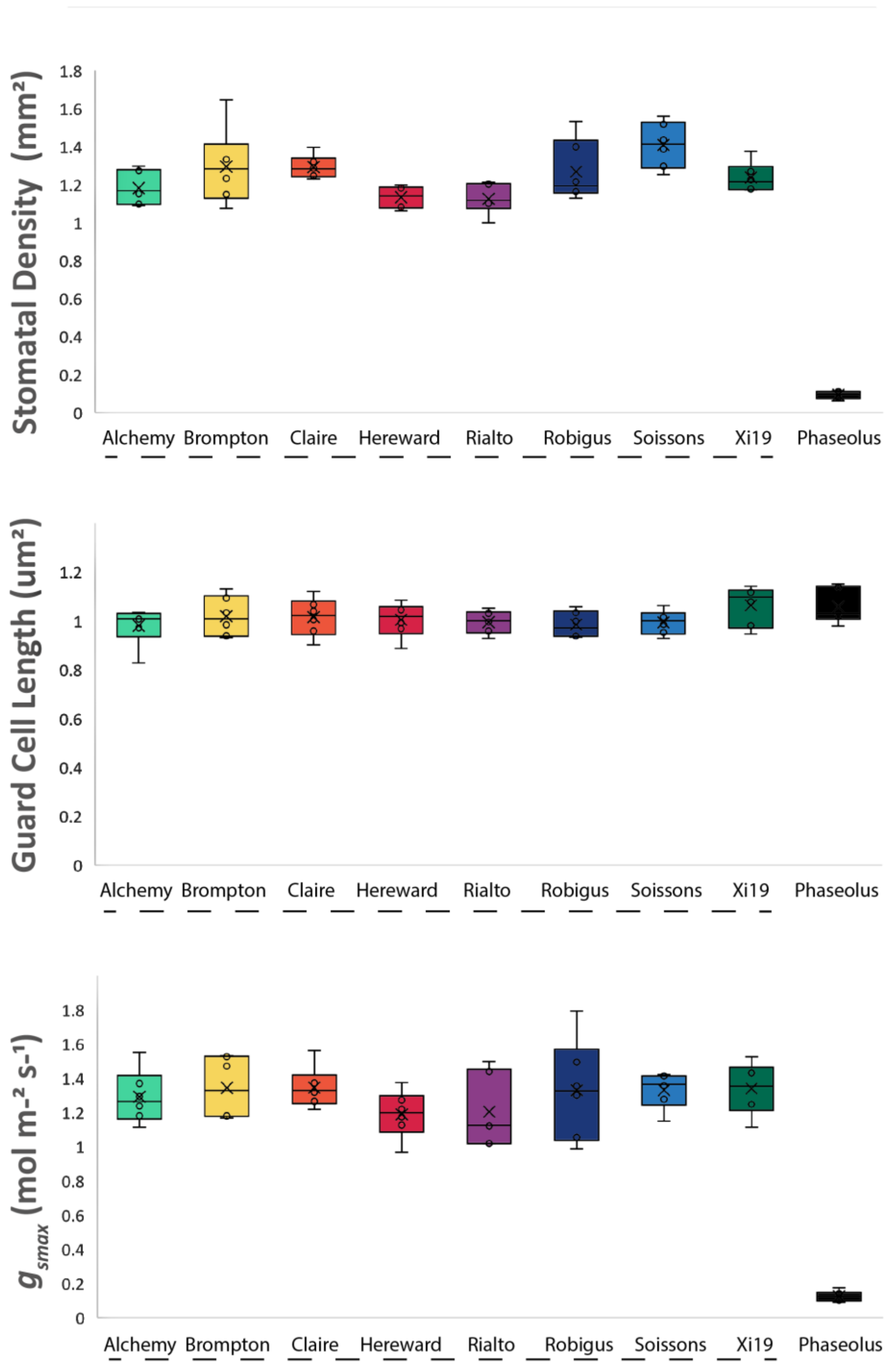

Fig. S9: Boxplots of stomatal anatomy ratios the abaxial and adaxial leaf surfaces in eight wheat (*Triticum aestivum*) cultivars (underlined in a broken line) and one French bean (*Phaseolus vulgaris)* cultivar. Variation (box and whisker plots displaying the distribution of biological replicates) and mean (black x) of SD ratios, GCL ratios and g_{smax} ratios between the abaxial and adaxial leaf surfaces. (n = 6).
